# Hierarchical Polyimide Nonwoven Fabric with Ultralow-Reflectivity Electromagnetic Interference Shielding and High-Temperature Resistant Infrared Stealth Performance

**DOI:** 10.1007/s40820-024-01590-3

**Published:** 2024-12-03

**Authors:** Xinwei Tang, Yezi Lu, Shuangshuang Li, Mingyang Zhu, Zixuan Wang, Yan Li, Zaiyin Hu, Penglun Zheng, Zicheng Wang, Tianxi Liu

**Affiliations:** 1 The Key Laboratory of Synthetic and Biological Colloids, Ministry of Education, School of Chemical and Material Engineering, International Joint Research Laboratory for Nano Energy Composites, Jiangnan University, Wuxi, 214122 Jiangsu People’s Republic of China; 2 Jiangsu Ferrotec Semiconductor Technology Co., Ltd., Yancheng, 214000 Jiangsu People’s Republic of China; 3 Guizhou Aerospace Wujiang Electro-Mechanical Equipment Co., Ltd., No. 20-5, Dalian Road Aerospace Industrial Park, Huichuan District, Zunyi City, 563000 Guizhou People’s Republic of China; 4 Civil Aircraft Fire Science and Safety Engineering Key Laboratory of Sichuan Province, College of Civil Aviation Safety Engineering, Civil Aviation Flight University of China, Guanghan, 618307 Sichuan People’s Republic of China

**Keywords:** Polyimide, Electromagnetic interference shielding, Low reflectivity, Infrared stealth, Compatibility

## Abstract

Designing and fabricating a compatible low-reflectivity electromagnetic interference (EMI) shielding/high-temperature resistant infrared stealth material possesses a critical significance in the field of military. Hence, a hierarchical polyimide (PI) nonwoven fabric is fabricated by alkali treatment, in-situ growth of magnetic particles and "self-activated" electroless Ag plating process. Especially, the hierarchical impedance matching can be constructed by systematically assembling Fe_3_O_4_/Ag-loaded PI nonwoven fabric (PFA) and pure Ag-coated PI nonwoven fabric (PA), endowing it with an ultralow-reflectivity EMI shielding performance. In addition, thermal insulation of fluffy three-dimensional (3D) space structure in PFA and low infrared emissivity of PA originated from Ag plating bring an excellent infrared stealth performance. More importantly, the strong bonding interaction between Fe_3_O_4_, Ag, and PI fiber improves thermal stability in EMI shielding and high-temperature resistant infrared stealth performance. Such excellent comprehensive performance makes it promising for military tents to protect internal equipment from electromagnetic interference stemmed from adjacent equipment and/or enemy, and inhibit external infrared detection.

**Supplementary Information:**

The online version contains supplementary material available at 10.1007/s40820-024-01590-3.

## Highlights

- Hierarchical polyimide (PI) nonwoven fabric is fabricated by alkali treatment, in-situ growth of magnetic particles, and "self-activated" electroless Ag plating process.
- Impedance matching structure by assembling Fe_3_O_4_/Ag-loaded PI nonwoven fabric (PFA) and pure Ag-coated PI nonwoven fabric (PA) induces more electromagnetic waves enter PFA/PA and be dissipated.
- The fluffy 3D space structure of PFA with strong adhesion interaction and low infrared emissivity of PA endow PFA/PA with excellent thermal stability in electromagnetic interference shielding and high-temperature resistant infrared stealth performance.

**Supplementary Information:**

The online version contains supplementary material available at 10.1007/s40820-024-01590-3.

## Graphical abstract

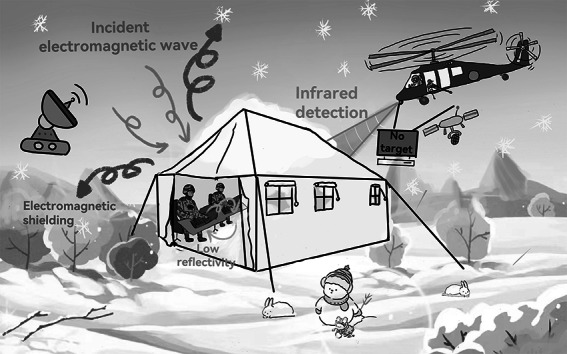

## Introduction

As the electronics and information industry rapidly develops, electromagnetic leakage and high-temperature infrared radiation become serious problems [1, 2]. Especially in the field of military protection, they easily lead to the information leakage and infrared exposure [3–6]. Hence, designing and fabricating compatible electromagnetic interference (EMI) shielding and infrared stealth material possesses a significant application value [2, 7, 8]. In comparison with those traditional metal and carbon-based nanomaterials, polymer-based composites attract increasing attention for EMI shielding and infrared stealth application, due to low density, corrosion resistance, low weight, and good processability [9–13].

Among them, metallized polymer nonwoven fabric gradually becomes a promising electromagnetic protection material, owning to its ultralight density, excellent flexibility, high electrical conductivity, and low infrared emissivity [14–17]. As reported [14], nickel-metallized polyimide nonwoven fabric is prepared by polydopamine roughening, palladium-free activation of chloroplatinic acid, electroless nickel plating, and octadecanethiol corrosion-resistant treatment. It exhibits a high electromagnetic interference shielding effectiveness (EMI SE) value of 68.6 dB, due to their high electrical conductivity (953 S cm^−1^). However, its higher electrical conductivity usually leads to the generation of impedance mismatch on interface between material and air. Most of incident electromagnetic waves (EMWs) are reflected to the original medium. As a result, the reflected EMWs are prone to induce a secondary pollution, severely interfering the normal operation of adjacent electronic devices. Meanwhile, the introduction of metal coating endows the composite with an excellent thermal conductivity. The higher thermal conductivity transmits lots of heat energy derived from self-heating electrical equipment, thereby inducing the formation of obvious infrared radiation. The undesired infrared radiation is easily detected by infrared detectors. Therefore, how to realize the synergistic design and fabrication of compatible composites with low-reflectivity EMI shielding and infrared stealth performance gradually becomes a big challenge in the system of metallized polymer nonwoven fabric.

Many explorations have been attempted to assembly the efficient low-reflectivity electromagnetic interference (EMI) shielding materials [18–22]. For example, Ma et al. successfully constructs multilayer CNF/MXene/FeCo composite film with controlled magnetic-conductivity dual-gradient structure by the layer-by-layer vacuum filtration method [19]. The composite film at a thickness of 340 μm exhibits an excellent EMI SE of 58.0 dB, whereas the corresponding *R* coefficient decreases to 0.61. However, a significant amount of EMW is still reflected due to its higher *R* coefficient, which needs to be further optimized. In addition, a high-performance infrared stealth material should be required to satisfy the Stefan–Boltzmann law: *E* = *εσΤ*^4^, where *E* is the infrared radiation intensity of material, *ε* is the infrared (IR) emissivity, *σ* is the Stefan–Boltzmann constant, and *Τ* is the surface temperature [23–25]. Therefore, an excellent infrared stealth performance can be achieved by reducing the IR emissivity and/or surface temperature of material. To reduce the IR emissivity, the surface electrical conductivity of materials should be increased, enhancing the reflection of mid-far (3–5 and 8–14 μm) infrared radiations. For example, as reported by Deng et al. [26], a highly densified and large-area MXene coating is prepared by micro-crosslinking between catecholamine and MXene. As a consequence, MXene coating presents low IR emissivity (0.179) and infrared stealth performance. However, the ultrahigh electrical conductivity of 12,247 S cm^−1^ leads to the formation of impedance mismatch, causing serious secondary electromagnetic pollution. On the other hand, porous insulating materials are used to reduce surface temperatures by blocking the thermal diffusion originated from the high-temperature heat sources. As reported by Wang et al. [27], an ultralight and mechanically robust polyimide curly nanofibrous aerogels are prepared by manipulated the solution/water (H_2_O) molecule interaction and ejection mode. As a result, those aerogels possess high porosity of 99.8% and excellent infrared stealth performance. However, the low permittivity and permeability of porous insulation materials are difficult to achieve effective dissipation of incident EMW, due to the existence of free space. To effectively dissipate the incident EMW, many functional nanofillers are introduced into porous materials. For example, Gu et al. successfully prepares a poly(3,4-ethylenedioxythiophene):poly(sodium styrenesulfonate)-coated melamine foam by dip-coating process [28]. As a result, the formation of porous structure in foam endows it with excellent infrared stealth (*ΔT* = 35.9 °C) and microwave absorption properties (*RL*_*min*_ = − 57.57 dB, d = 5 mm). However, the thinker thickness significantly reduces the convenience of material. Moreover, the functional nanofiller for electromagnetic dissipation in porous insulation materials is generally introduced by in-situ compounding or vacuum impregnation methods. Nevertheless, the in-situ composite approach inevitably reduces the mechanical properties of composite. Additionally, although vacuum impregnation can be employed to build an electromagnetic dissipation network without altering mechanical properties, the weaker bonding force between the functional nanofiller and skeleton trough van der Waals interaction make it easy for the functional nanofiller to strip from skeleton, which seriously limits their durability in the high-temperature environments [29]. Therefore, it is difficult to achieve the integrated construction of electromagnetic interference shielding and high-temperature infrared stealth using only one material. How to realize the compatibility of low-reflectivity EMI shielding and high-temperature resistant infrared stealth becomes the huge challenge that needs to be solved urgently.

In this paper, a hierarchical polyimide (PI) nonwoven fabric with ultralow-reflectivity EMI shielding and high-temperature resistant infrared stealth performance are prepared by alkali treatment, in-situ growth of magnetic particles and "self-activated" electroless silver plating process. The alkali treatment facilitates the hydrolyzation of imide rings in PI molecules, thereby leaving abundant carboxylic active sites. Those carboxylic active sites can be served as strong bonding anchors for Ag nanoparticles generated by Ag^+^ exchange and chemical reduction. It facilitates the effective "self-activated" deposition of Ag atoms during electroless silver plating process, leading to the formation of pure Ag-coated PI nonwoven fabric (PA). In addition, those carboxylic active sites provide strong adhesion for in-situ growth of magnetic particles (Fe_3_O_4_) through complexation with iron ions (Fe^3+^) and annealing treatment. Subsequently, to further increase the quantity of active sites, dopamine (DA) can be polymerized on the surface of Fe_3_O_4_-loaded PI nonwoven fabric (PF) as a strong adhesion roughened coating. The catechol and amine functional groups remaining in polydopamine can further act as ligand/reduction agent for Ag^+^. Those obtained Ag nanoparticles can accelerate the effective "self-activated" deposition of Ag atoms during electroless silver plating process. As a consequence, the impedance characteristic of Fe_3_O_4_/Ag-loaded PI nonwoven fabrics (PFA) can be easily adjusted by controlling the in-situ growth of magnetic particles and "self-activated" electroless silver plating process. The synergistic fabrication of PFA and PA facilitates the rational construction of hierarchical impedance matching in PFA/PA. It induces more EMW enter the composite and be dissipated as much as possible, endowing it with an ultralow-reflectivity EMI shielding performance. Moreover, thermal insulation of fluffy 3D space structure in PFA and IR emissivity of PA originated from Ag plating bring a superior infrared stealth performance. More importantly, the strong adhesion interaction between Fe_3_O_4_, Ag, and PI fiber allows it resist the thermal stress derived from high-temperature source, enhancing the thermal stability in EMI shielding and high-temperature resistant infrared stealth performance. Such excellent compatible ultralow-reflectivity EMI shielding/infrared stealth performance makes it possible for PFA/PA to become a potential candidate with competitive advantage in military tent and/or camouflage.

## Experimental Section

### Materials

Tris(hydroxymethyl) aminomethane (Tris, ≥ 99%), dopamine (DA, ≥ 99%), dimethylaminoborane (DMAB, ≥ 97%), D-( +)-glucose (≥ 99%), L-potassium sodium tartrate (≥ 99%), and iron(III) chloride hexahydrate (FeCl_3_·6H_2_O, ≥ 99%) were purchased from Adamas Reagent Co., Ltd. Ethanol (≥ 99.7%), ammonium hydroxide aqueous solution (NH_3_·H_2_O, 25% ~ 28%), silver nitrate (AgNO_3_, ≥ 99.8%), and sodium hydroxide (NaOH, ≥ 96%) were purchased from Sinopharm Chemical Reagent Co., Ltd. 75 spray glue was purchased from Minnesota Mining and Machinery Company. All other chemicals were used without further purification as received.

### Preparation of Ag-Coated Polyimide Nonwoven Fabrics

The hot-pressed and compact PI nonwoven fabric was prepared according to our previously reported method [30]. Subsequently, the compact PI nonwoven fabric was immersed in a 10 wt% NaOH solution for 3 min. Then, the obtained nonwoven fabric was sufficiently washed by deionized (DI) water. Afterward, the obtained nonwoven fabric was placed in 5 g L^−1^ AgNO_3_ and 2 g L^−1^ DMAB solution to be immersed for 2 and 0.5 h. Finally, the nonwoven fabric was fully washed with DI water and placed in an electroless Ag plating solution for x h (marked as PA_x_). For electroless Ag plating solution, AgNO_3_ was dissolved in deionized water, NH_3_·H_2_O was added until the solution was clarified to obtain silver-ammonia solution. And then 50 mL of solution containing 80 g L^−1^ glucose, 2.5 g L^−1^ potassium sodium tartrate, and 100 g L^−1^ ethanol was added to the silver-ammonia solution.

### Preparation of Fe_3_O_4_/Ag-Loaded Polyimide Nonwoven Fabrics

First, a fluffy PI nonwoven fabric was prepared according to our previously reported method [14, 31]. Subsequently, the nonwoven fabric was immersed in a 10 wt% NaOH solution for 3 min. Afterward, the obtained nonwoven fabric was washed sufficiently by DI water to remove residual NaOH. Then, the obtained nonwoven fabric was placed in a 37 wt% solution of FeCl_3_·6H_2_O at 80 °C for 5 h. Afterward, the treated PI nonwoven fabric was washed by anhydrous ethanol and then placed in an oven at 80 °C for 2 h. Finally, the dried PI nonwoven fabric was treated in a tube furnace and heated up to 400 °C at a rate of 5 °C min^−1^ in an atmosphere of 12 vol% hydrogen-argon (H_2_/Ar) mixture. Afterward, the nonwoven fabric was treated at 400 °C for 4 h. As a results, magnetic nanoparticle-coated PI fiber (PF) was obtained. PF nonwoven fabric was then placed in 10 mM Tris solution at pH = 8.5. 2 g L^−1^ DA was added, and the reaction was carried out under magnetic stirring for 24 h. After the reaction, the obtained nonwoven fabric was washed sufficiently with DI water. And then it was placed in 5 g L^−1^ AgNO_3_ to be immersed for 2 h. Afterward, the nonwoven fabric was fully washed with DI water and placed in a electroless Ag plating solution for y h (marked as PFA_y_). Therefore, PF can be also labeled as PFA_0_.

### Preparation of Hierarchical Polyimide Composite Nonwoven Fabrics

The hierarchical polyimide nonwoven fabric was prepared by combining PA_x_ with different thicknesses of PFA_y_. Finally, the nonwoven fabric was pasted by 75 spray glue, which was denoted as PFA_y_/PA_x_ (or PFA/PA if not emphasized).

### Characterizations

The morphology of the specimens was observed by a field emission scanning electron microscope (FE-SEM) at an accelerating voltage of 30 kV. Energy-dispersive X-ray spectroscopy (EDS) analysis was also performed to analyze the surface composition of materials. X-ray diffraction (XRD) pattern was characterized on a Bruker D2 X-ray diffractometer with a Cu K*α* X-ray source (*λ* = 1.5418 Å). X-ray photoelectron spectroscopic (XPS) was conducted on an ESCA 2000 (VG Micro-Tech, UK) using a monochromic Al Kα X-ray source. Thermal gravimetric analysis (TGA) was carried out using a TGA/DSC1/1100SF system under a N_2_ atmosphere at a heating rate of 10 °C min^−1^. Polyimide nonwoven fabric was prepared by electrostatic spinning machine (ET-2535H, Beijing Ucalery Co., Ltd., China). The sheet resistance (*R*) of nonwoven fabric was measured by the four-probe method with a ST2263 double electric measuring digital four-probe tester at room temperature. The electrical conductivity (*σ*) was obtained by the Eq. 1 [32]:

1 $$ \sigma = \frac{1}{S} \cdot \frac{1}{R/L} = \frac{L}{R \cdot w \cdot t} $$

where *σ* was the electrical conductivity (S cm^−1^), *R* was the sheet resistance (Ω sq^−1^), *L* and *S* were the length (cm) and cross-sectional area (cm^2^) of sample, and *w* and* t* were the width (cm) and thickness (cm). Using Agilent E5234B vector network analyzer and waveguide method, EMI SE was measured in X-band (8.2–12.4 GHz), Ku-band (12.4–18 GHz), K-band (18–26.5 GHz), and Ka-band (26.5–40 GHz). The corresponding EMI SE (SE or SE_T_) can be obtained by calculating the scattering parameters (S_11_ and S_21_) as follow Eqs. 2–7 [33, 34]:

2 $$ R = |S_{11} |^{2} = |S_{22} |^{2} $$

3 $$ T = |S_{21} |^{2} = |S_{12} |^{2} $$

4 $$ A = 1 - R - T $$

5 $$ {\text{SE}}_{R} = - 10\log \left( {1 - R} \right) = - 10\log \left( {1 - \left| {S_{11} } \right|^{2} } \right) $$

6 $$ {\text{SE}}_{A} = - 10\log \left( {\frac{T}{1 - R}} \right) = - 10\log \left( {\frac{{\left| {S_{21} } \right|^{2} }}{{1 - \left| {S_{11} } \right|^{2} }}} \right) $$

7 $$ {\text{SE}}_{T} = {\text{SE}}_{R} + {\text{SE}}_{A} + {\text{SE}}_{M} $$

when the SE_T_ exceeded 10 dB, SE_M_ can be generally negligible. Reflectivity values (dB) were calculated by the measured S_11_ according to the Eq. 8, which denoted the microwave absorption performance of the nonwoven fabrics for incident EMW [35, 36].

8 $$ {\text{Reflectivity}} = 10 \log R = 10 \log |S_{11} |^{2} $$

Furthermore, a self-made electromagnetic wave transmitter–receiver (10.5 GHz, 1W) and a wireless projector (PeakDo, 60 GHz, Shenzhen Xiwei Digital Technology Co., Ltd.) were carried out to demonstrate the EMI shielding performance of samples. Paperless recorder with thermocouple (MIK-R2007, Hangzhou Miko sensor Co., Ltd.) was applied to record the changes in the surface temperature of samples. The nonwoven fabric was placed on a thermal stage (100 mm*100 mm, Shenzhen Jinglianghe Technology Co., Ltd.) to evaluate the infrared stealth performance (FLUKE Ti400 + , Fluke Corporation of the USA). The IR emissivity (*ε*) was calculated by Eq. 9 after testing the reflectivity (*r*) and transmissivity (*t*) [37].

9 $$ \varepsilon = 1 - r - t $$

The *t* and *r* were obtained from Fourier transform infrared spectroscopy (FTIR, Nicolet IS50, Thermo Fisher Scientific Inc., USA) with an integrating sphere.

## Results and Discussion

### Structure and Morphology of Composite Nonwoven Fabrics

**Scheme 1:**
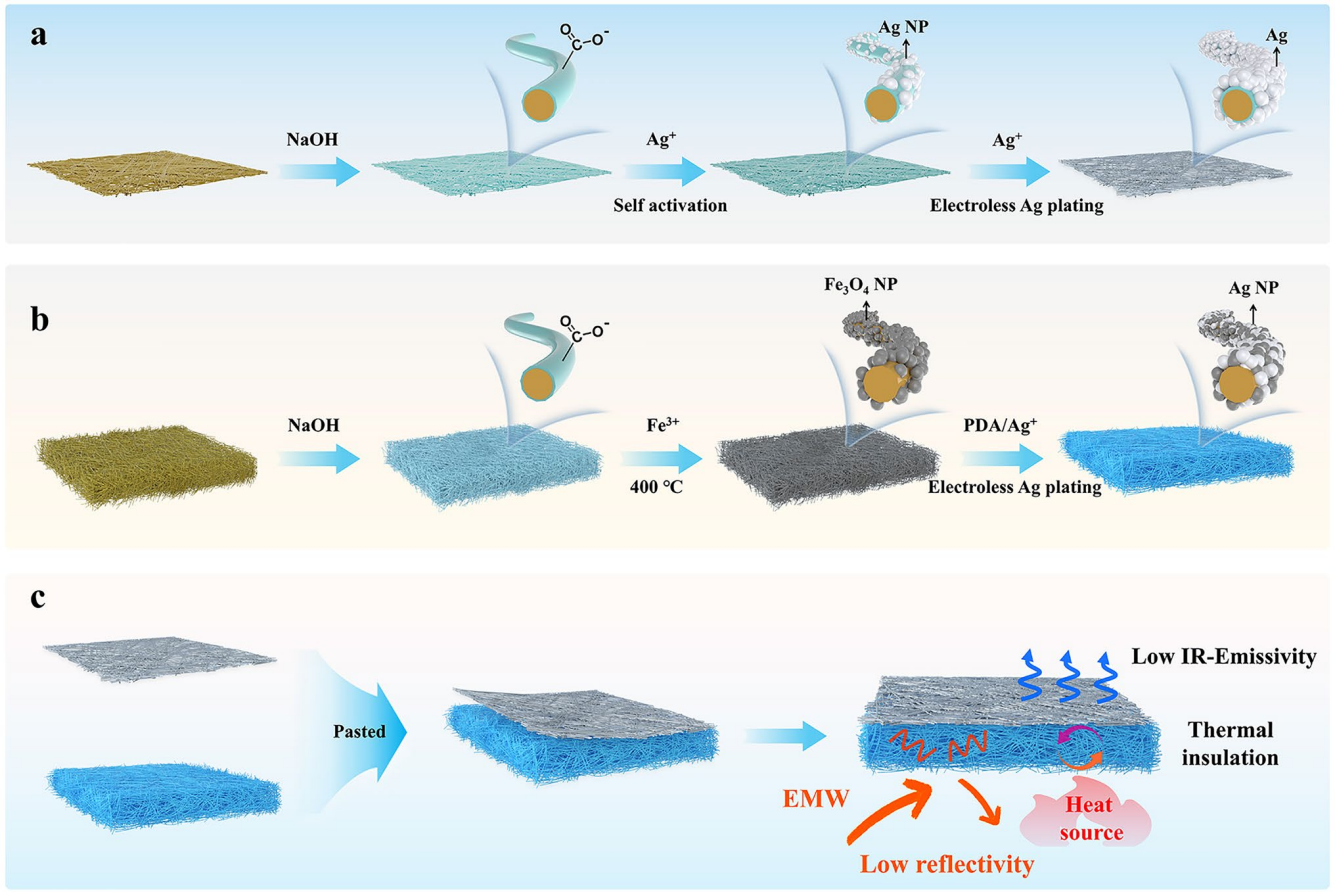
Schematic illustration of the preparation process of PFA/PA nonwoven fabrics

**Fig. 1:**
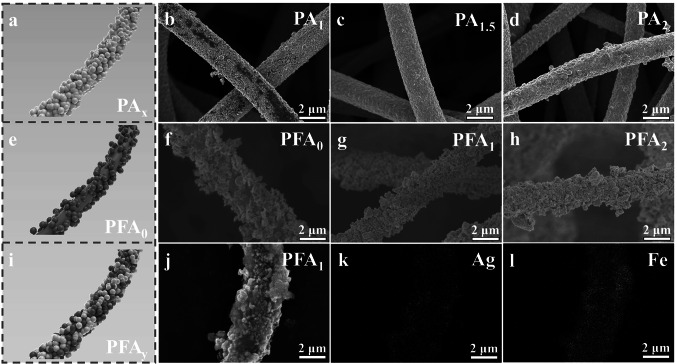
Schematic illustration of **a** PA_x_, **e** PFA_0_, and **i** PFA_y_. SEM images of **b** PA_1_, **c** PA_1.5_, **d** PA_2_, **f** PFA_0_, **g** and **j** PFA_1_, **h** PFA_2_ fiber, and **k, l** EDS mapping images of PFA_1_ fiber

**Fig. 2:**
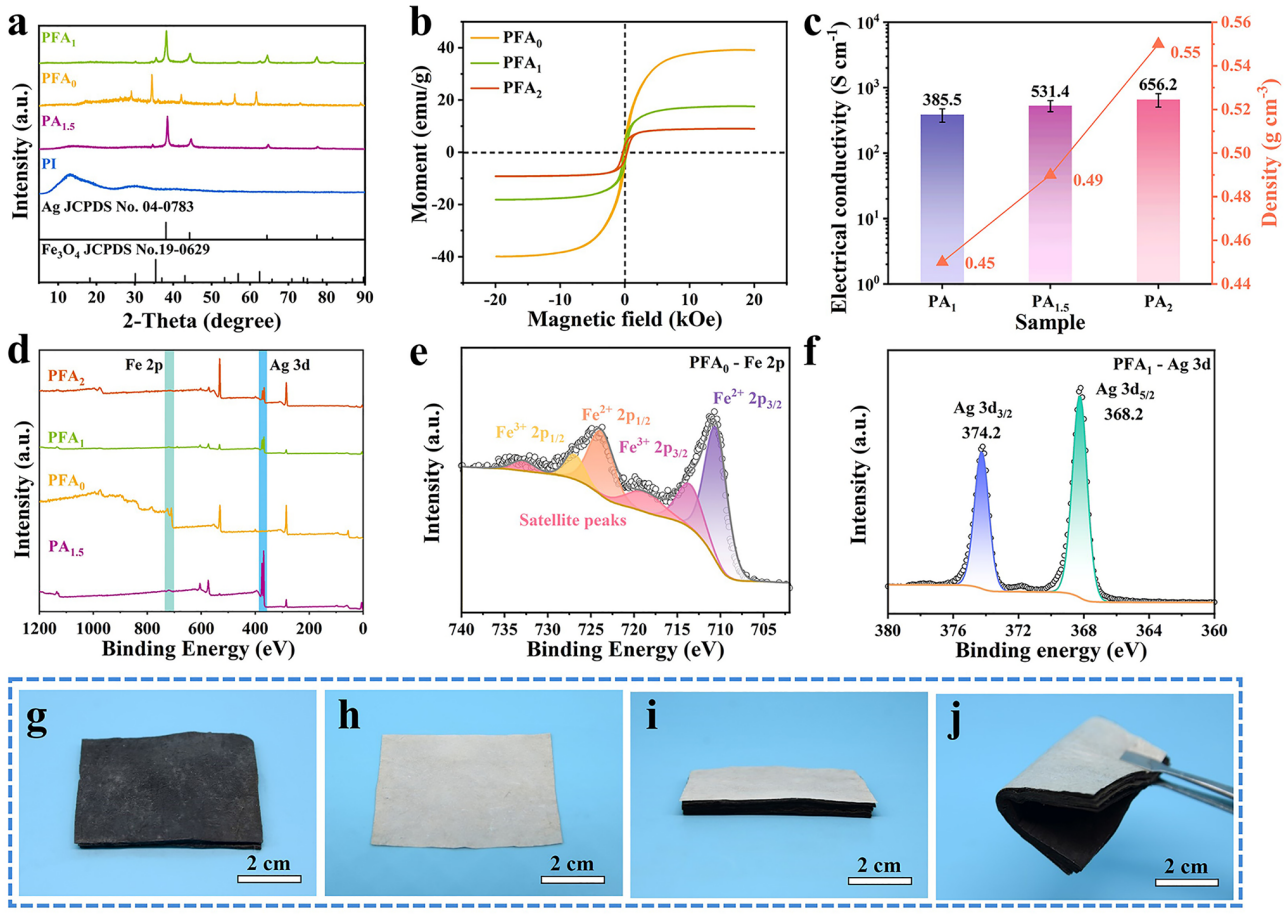
**a** XRD patterns of PI, PA_1.5_, PFA_0_, and PFA_1_. **b** Hysteresis loops of PFA_0_, PFA_1_, and PFA_2_. **c** Electrical conductivity and density of PA_1_, PA_1.5_, and PA_2_. **d** XPS spectra of PA_1.5_, PFA_0_, PFA_1_, and PFA_2_. High resolution XPS spectra of Fe 2*p* for **e** PFA_0_ and **f** Ag 3*d* for PFA_1_. Optical photograph of **g** 4*PFA_1_, **h** PA_1.5_, and **i**, **j** 4*PFA_1_/PA_1.5_

The fabrication process of hierarchical polyimide (PFA/PA) nonwoven fabrics is demonstrated in Scheme 1. Firstly, PI nonwoven fabrics are prepared by a reported previous method [14, 31]. As we know, polyimide possesses high surface inertness [38–40]. Hence, the weaker bonding force between the functional nanofiller and PI fiber makes it easy for the functional nanofiller to exfoliate from polyimide fiber. To increase the surface activity of PI fiber, alkali etching treatment is applied by NaOH solution to promote the hydrolyzation of imide ring on the surface of PI fiber, thereby leaving abundant carboxyl active sites [41, 42]. As shown in Fig. S1a, c, the original PI fiber displays a smooth surface. After NaOH treatment, the obtained fiber presents a distinctly rough surface as displayed in Fig. S1b, d. It confirms the effectiveness of alkali etching. Based on the above, those carboxylic active sites can be act as strong bonding "chemical" anchors for Ag nanoparticles generated by Ag^+^ exchange and chemical reduction. As shown in Fig. S2, Ag nanoparticles with a diameter of ~ 30 nm can be effectively reduced by DMAB and uniformly deposited to the surface of PI fiber. More importantly, those Ag nanoparticles can be further acted as the "self-activated" center to accelerate the deposition rate of Ag atom during the electroless Ag plating process [43]. As shown in Fig. 1a–d, a compact plating can be successfully formed on the surface of PI fiber. Meanwhile, the plating degree of Ag can be further optimized as the electroless plating time increases. Specifically, the obtained Ag could not even cover the surface of PI fibers, as the plating time is less than 1 h (Fig. 1b). Some blanks/defects leave on PA_1_. Those blanks/defects can be effectively compensated when the plating time is prolonged to 1.5 h. The corresponding surface of PA_1.5_ becomes smoother (Fig. 1c). When the plating times are further prolonged to 2 h, the surface of PA_2_ becomes rough, which may be attributed to the excess Ag plating (Fig. 1d). It is inevitable that the undesired aggregation of Ag nanoparticles would lead to increase in roughness of fiber. Meanwhile, it can be distinctly observed that the color of nonwoven fabric transfers from bright yellow of PI to silver-white (Fig. 2h) after electroless Ag plating. It indirectly indicates the effectiveness of Ag deposition. To further demonstrate the successful preparation of modified polyimide composites, the nonwoven fabric is also characterized by X-ray diffraction spectra (XRD). As displayed in Fig. 2a, a visible characteristic diffraction peak located at 38° arise in curve of PA_1.5_ in compared with that of PI. It can be assigned to the plane (111) of face-centered cubic phase of Ag. In order to further probe the surface chemical change, those nonwoven fabrics are characterized by X-ray photoelectron spectroscopy (XPS). As show in Fig. 2d, a new peak of Ag elements located at 368.8 eV appears in PA_1.5_. Based on the above, it can be concluded that Ag is successfully loaded on the surface of PI fiber by “self-activated” electroless Ag plating. In addition, the electrical conductivity of PA_x_ is measured and recorded. As shown in Fig. 2c, it presents a progressive tendency to increase from 385 (1 h) to 531.4 S cm^−1^ (1.5 h), and then to 656.2 S cm^−1^ (2 h) with the increasing the electroless Ag plating time. Therefore, the adjustable impedance characteristic of PA_x_ can be endowed by the different electrical conductivities. Moreover, the corresponding density exhibits a similar tendency increase from 0.45 to 0.55 g cm^−3^ (Fig. 2c), which demonstrates the PA_x_ nonwoven fabric possesses a light characteristic.

Moreover, those carboxylic active sites can provide strong adhesion for in-situ growth of magnetic particles (Fe_3_O_4_) through complexation with iron ions (Fe^3+^) and annealing treatment. Specifically, the high temperature (400 °C) and H_2_ can act as a reducing agent for Fe^3+^ during annealing treatment in H_2_/Ar atmosphere. During annealing treatment, a part of Fe^3+^ can be reduced to Fe^2+^. As a result, Fe_3_O_4_ nanoparticle can be prepared by annealing treatment in H_2_/Ar atmosphere [44]. As demonstrated in Fig. 1e, f, Fe_3_O_4_ nanoparticles are successfully loaded on the surface of PI fiber. The corresponding energy-dispersive spectrometer (EDS) results demonstrate that Fe element is successfully distributed on the surface of PI fiber (Fig. S3). Similarly, XRD is utilized to analyze the crystalline structure of magnetic PI-based nonwoven fabric (PFA_0_). In comparison with PI, a series of new diffraction peaks for PFA_0_ appears at 30°, 35°, 43°, 53°, 56°, and 62° as described in Fig. 2a. Those diffraction peaks correspond to the indexed planes of Fe_3_O_4_ (JCPDS No. 19-0629), which is indirectly confirm the formation of Fe_3_O_4_ on the surface of PI fiber. Furthermore, the high-resolution XPS spectra of Fe 2*p* for PFA_0_ can be differentiated into four characteristic peaks to Fe 2*p*_3/2_ and Fe 2*p*_1/2_ at 710.8, 713.9, 724.2, and 727.3 eV, respectively (Fig. 2e). Meanwhile, those two satellite peaks of Fe 2*p*_3/2_ and Fe 2*p*_1/2_ appear at 719.1 and 733.1 eV. It indicates that the valence state of Fe in PFA_0_ is Fe^3+^ and Fe^2+^ [45]. High resolution XPS spectra of O 1*s* of PFA_0_ are also given in Fig. S4. Two peaks located at 530.0 and 531.5 eV respond to Fe–O and C=O. Those results once again confirm the formation of Fe_3_O_4_ on the surface of PI fiber. However, the exposed area of PI fibers is reduced, due to the loading of Fe_3_O_4_ nanoparticles covered on the surface of PI fibers. PFA_0_ fiber possesses less sites to be treated by NaOH, thereby further hindering the achievement of subsequent "self-activated" electroless Ag plating. As a result, it is difficult to realize the effective adjustability in impedance characteristic of PFA. Hence, dopamine (DA) with abundant catechol and amine functional groups is employed to increase the quantity of active sites as a roughening agent, which can be polymerized on the surface of PFA_0_ fiber. The abundant hydroxyl groups on the surface of PDA will give PFA_0_ fibers excellent hydrophilicity [37]. As shown in Fig. S5, the water contact angle transforms from 125.8° (PFA_0_) to 29.6° (PFA_0_/PDA). It indirectly indicates that PDA is successfully polymerized on the surface of PFA_0_ fibers. Very importantly, the obtained PDA coating also possesses a strong adhesion, due to the residual catechol and amine functional groups in PDA. It provides a strong bonding force at the interfaces between subsequent Ag nanoparticles and PFA_0_ fibers [46, 47]. Additionally, the residual catechol and amine functional groups in PDA molecules endow PDA with an excellent adsorption and reduction capabilities for Ag^+^ [48]. As a result, PDA coating can also act as ligand/reduction agent to facilitate the in-situ immobilization/reduction of Ag^+^ to Ag nanoparticle. Those Ag nanoparticles can serve as a "self-activated" center to accelerate the reduction reaction of Ag atom during electroless silver plating. As shown in Fig. 1f, the obtained Fe_3_O_4_ nanoparticle even can’t cover the whole surface of PI fiber, when the electroless Ag plating is not applied. Some gaps/defects leave on the surface of PI fiber. As the electroless Ag plating time is increased to 1 h, the surface of PFA_1_ becomes rougher (Fig. 1g). As shown in a higher magnification SEM image of PFA_1_ (Fig. S6), Ag nanoparticles show a smaller diameter compared to that of Fe_3_O_4_ particles. Ag nanoparticles effectively compensate those gaps/defects left from Fe_3_O_4_, and cover the original Fe_3_O_4_. When the electroless Ag plating time is prolonged to 2 h, more Ag nanoparticles are deposited, leading to an increase in the roughness of PFA_2_ (Fig. 1h). Moreover, for PFA_1_, the plane (111) of face-centered cubic phase of Ag appears at 38° as compared to PFA_0_ (Fig. 2a). It indicates that Ag is successfully deposited on the surface of PFA_1_ by electroless Ag plating. EDS is also employed to characterize the elements changes in PFA_x_ fiber before and after electroless Ag plating. As shown in Figs. 1j–l and S7, Ag element displays a uniform and distinct distribution on the surface of PFA_1_ fiber, compared to the PFA_0_ (Fig. S3). It once again confirms that Ag is successfully loaded on the surface of PFA_1_ fiber. In addition, to further probe the surface chemical change, those nonwoven fabrics are characterized by XPS. As shown in Fig. 2d, Fe elements exist in PFA_0_, PFA_1_, and PFA_2_. Compared with PFA_0_, the characteristic peak of Fe 2*p* significantly reduces in XPS curves of PFA_1_ and PFA_2_. It may be ascribed to the fact that Fe_3_O_4_ is gradually covered by Ag with the increasing content of electroless Ag plating. Meanwhile, vibrating-sample magnetometer (VSM) is employed to characterize the evolution in magnetic properties of PFA_y_ under different electroless Ag plating times. Compared with PFA_0_, the saturation magnetization (Ms) gradually decreases with increasing plating time as shown in Fig. 2b. In detail, PFA_0_ exhibits a high Ms value of 39.20 emu g^−1^, while that of PFA_1_ and PFA_2_ decreases to 17.64 to 9.05 emu g^−1^, respectively. It can be also ascribed to the increasing content of electroless Ag plating, resulting in a corresponding decrease in the proportion of Fe_3_O_4_ in PFA_y_. More importantly, the nonwoven fabrics do not undergo significant thermal decomposition until 400 °C (Fig. S8). It indicates that those nonwoven fabrics possess excellent thermal stability after in-situ growth of magnetic particles and "self-activated" electroless Ag plating process. In addition, the residual yield of PA_1.5_, PFA_0_, and PFA_1_ nonwoven fabrics is significantly higher than that of pure PI at higher temperature of 1000 °C, which once again confirms the excellent thermal stability of PFA. It can be attributed to the passivation of Fe_3_O_4_ and Ag plating formed on the surface of PI fibers. Besides, as shown in Fig. 2f, the Ag 3*d* region of PFA_1_ can be further divided into Ag 3*d*_5/2_ (368.2 eV) and Ag 3*d*_3/2_ (374.2 eV). Both of them can be recognized as Ag^0^ species [49]. Such results once again indicate that Ag is successfully obtained on PFA_0_ by electroless Ag plating. As a consequence, the final PFA_y_ nonwoven fabric presents a uniform black in color (Fig. 2g).

As a conclusion, the impedance characteristic of nonwoven fabrics can be easily adjusted by controlling the in-situ growth of magnetic particles and "self-activated" electroless Ag plating process. The synergistic fabrication of Fe_3_O_4_/Ag-loaded PI nonwoven fabric (PFA_y_) and pure Ag-coated PI nonwoven fabric (PA_x_) facilitates the construction of hierarchical impedance matching in PFA_y_/PA_x_. As shown in Fig. 2i, four layers of PFA_1_ with a thickness of 2.4 mm (4*PFA_1_) and a layer of PA_1.5_ with a thickness of 0.06 mm can be easily bonded by spray glue. The obtained 4*PFA_1_/PA_1.5_ exhibits color difference in positive and negative surfaces. Importantly, 4*PFA_1_/PA_1.5_ possesses an excellent flexibility. It can be bent at a wide angles (Fig. 2j), which is adapt to complex environments as an excellent flexible material.

### Ultralow-Reflectivity EMI Shielding Performance of Nonwoven Fabrics

As we all known, the construction of electromagnetic matching facilitates the introduction of EMW enter the interior of nonwoven fabric and be dissipated as much as possible, thereby endowing it with an excellent low-reflectivity EMI shielding performance. To clarify the role of PFA_y_ in the electromagnetic matching structure, PFA_1_ with different thicknesses (N*PFA_1_) is obtained by the same approach to further investigate their EMI shielding performance. The electromagnetic *S* parameters of those samples are measured in the frequency range of 8.2–12.4 GHz. Based on the electromagnetic *S* parameters, the final SE_T_, SE_A_, and SE_R_ can be calculated according to Eqs. 2–7. As displayed in Fig. S9a, SE_T_ of PFA_1_ exhibits an obvious tendency to increase form 2.1 dB (0.6 mm) to 3.9 dB (1.2 mm), 4.9 dB (1.8 mm), and then to 6 dB (2.4 mm) with the increasing thickness. However, they are much lower than that of commercial requirement (> 20 dB). In order to further clarify the EMI shielding mechanism, power coefficients of those samples can be calculated by Eqs. 2–4. As shown in Fig. S9b–d, the corresponding *A* and *R* coefficients exhibit a similar tendency to increase as the thickness of PFA_1_ increases, whereas *T* coefficient displays an opposite case. Especially, *T* coefficient of 1*PFA_1_ with a thickness of 0.6 mm is up to a high value (> 0.5) in X-band. It suggests that most of incident EMW penetrates the thin sample. When the thickness of PFA_1_ increases, the corresponding *T* coefficient decreases. It may be attributed to a fact that the increasing thickness of PFA_1_ effectively promotes the prolongation of EMW dissipation route, thereby endowing it with an increasing *A* coefficient and a decreasing *T* coefficient. Even so, the final *T* coefficient of 4*PFA_1_ is still maintained at a value of ~ 0.2. To further reduce the *T* coefficient, a high-reflectivity EMI shielding material should be introduced into the system.

**Fig. 3:**
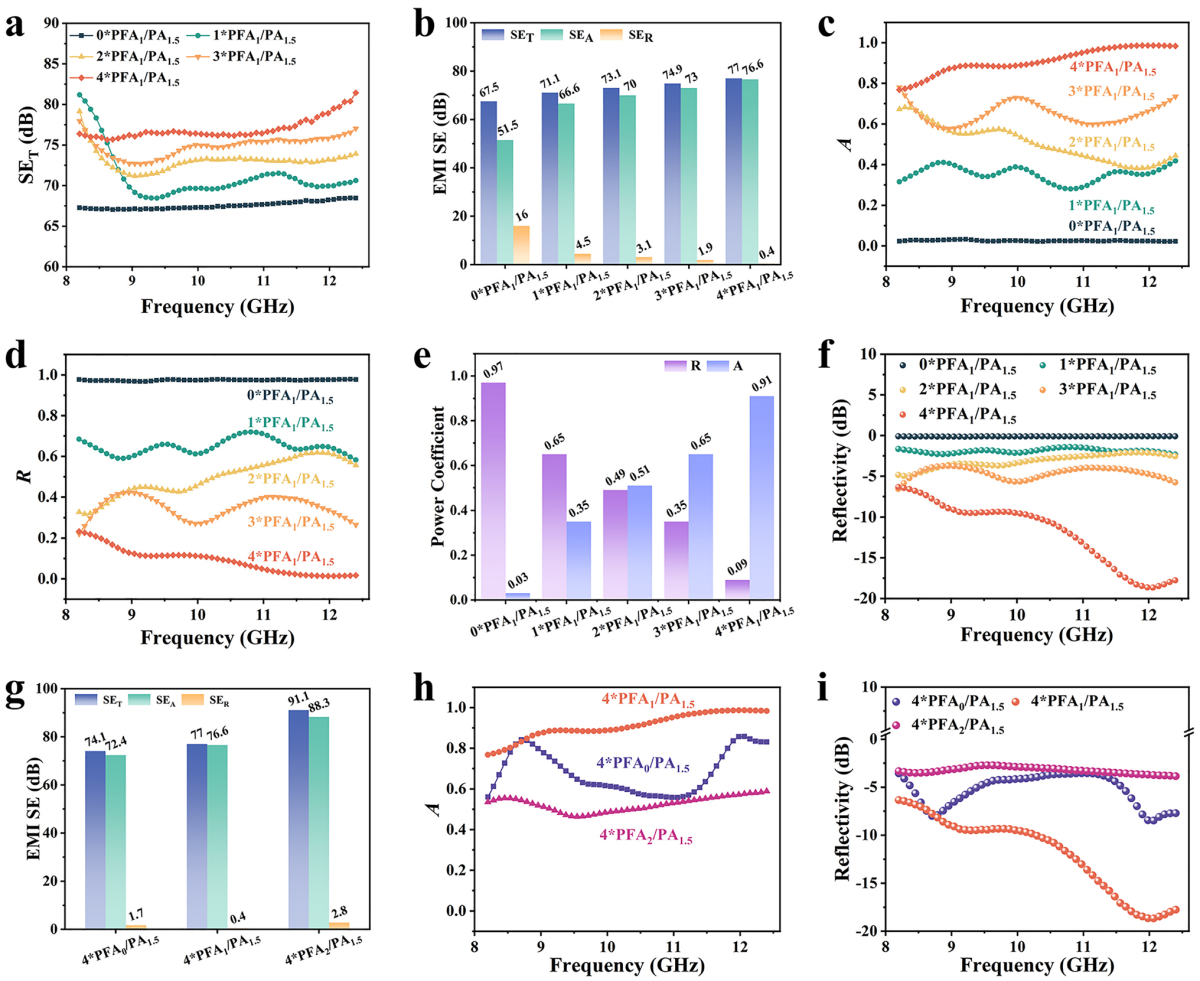
**a** SE_T_, **b** EMI SE, **c** *A*, **d** *R*, **e** power coefficient, **f** reflectivity patterns of N*PFA_1_/PA_1.5_, **g** EMI SE, **h** *A*, and **i** reflectivity patterns of 4*PFA_x_/PA_1.5_

Therefore, PA_1.5_ (0.06 mm) with a high electrical conductivity (531.4 S cm^−1^) is adhered on the backside of N*PFA_1_. As a control, the pure 0*PFA_1_/PA_1.5_ nonwoven fabric (PA_1.5_) possesses a high EMI SE value of 67.5 dB in X-band. Based on the above, EMI SE performance of N*PFA_1_/PA_1.5_ can be further enhanced to a higher value (> 67.5 dB) as displayed in Fig. 3a, b. It once again confirms the effectiveness of PA_1.5_ as high-reflectivity EMI shielding material. Meanwhile, EMI SE curve of N*PFA_1_/PA_1.5_ exhibits a tendency to increase from 71 to 77 dB with the increasing thickness of PFA_1_. It agrees well with the above result in Fig. S9. To further investigate the difference of EMI shielding mechanism, SE_A_, SE_R_, and power coefficients are also calculated and demonstrated in Fig. 3b, e. It is worthy to note that SE_R_ of N*PFA_1_/PA_1.5_ decreases from 16 to 0.4 dB with the increasing thickness of PFA_1_. Meanwhile, the corresponding *A* and* R* coefficient of N*PFA_1_/PA_1.5_ exhibit an opposite tendency. In detail, *A* coefficient increases from 0.03 to 0.35, to 0.51, to 0.65, and then to 0.91 with the increasing the thickness of PFA_1_ from 0 to 2.4 mm (Fig. 3c, e). The corresponding *R* coefficient decreases from 0.97 to 0.65, to 0.49, to 0.35, and then to 0.09. It may be attributed to a fact that the increasing thickness of PFA_1_ effectively prolongs the dissipation route of incident EMW. To further evaluate the dissipation capacity of N*PFA_1_/PA_1.5_ for EMW, reflectivity (dB) is calculated by Eq. 8. As displayed in Fig. 3f, reflectivity of N*PFA_1_/PA_1.5_ in X-band presents a tendency to decrease as the thickness of PFA_1_ increases. In detail, 0*PFA_1_/PA_1.5_ exhibits a high reflectivity (~ 0 dB). It suggests that almost EMW are reflected on the surface of 0*PFA_1_/PA_1.5_. The corresponding reflectivity of N*PFA_1_/PA_1.5_ decreases from ~ − 2 to ~ − 12 dB, as the thickness of PFA_1_ increases from 0.6 to 2.4 mm. It may be also explained by the increasing thickness of PFA_1_. It can effectively lengthen the dissipation route of EMW, resulting in an increasing absorption and a decreasing reflection for EMW. More importantly, reflectivity of 4*PFA_1_/PA_1.5_ is lower than -10 dB in 10.3–12.4 GHz. It indicates that more than 90% of incident EMW can be absorbed in 4*PFA_1_/PA_1.5_. Therefore, 4*PFA_1_/PA_1.5_ exhibits a huge promise to be employed as a superior radar stealth material in 10.3–12.4 GHz.

As we all known, the impedance characteristic of samples are the key factors for fabricating low-reflectivity EMI shielding materials. Those EMI SE and power coefficients of PFA_x_ with different electroless Ag plating times are presented in Fig. S10. Without Ag plating, 4*PFA_0_ exhibits an ultralow EMI SE_T_ value of 1.9 dB in X-band. When the electroless Ag plating time is prolonged to 1 and 2 h, the corresponding SE_T_ of 4*PFA_x_ increases to 6 and 23 dB (Fig. S10a), respectively. Meanwhile, *T* coefficient of 4*PFA_x_ displays an outstanding tendency to decrease from ~ 0.65 (4*PFA_0_) to ~ 0.25 (4*PFA_1_), and then to ~ 0 (4*PFA_2_), as shown in Fig. S10d. It can be attributed to the increase in conductive Ag nanoparticle content, resulting in the increase in absorption and reflection capacity of PFA_x_. In detail, *R* coefficient exhibits a prominent increase from ~ 0.01 (4*PFA_0_) to ~ 0.2 (4*PFA_1_), and then to 0.44 (4*PFA_2_), as displayed in Fig. S10c. As a result, the corresponding *A* coefficient varies from ~ 0.3 (4*PFA_0_) to ~ 0.53 (4*PFA_1_) and maintains at ~ 0.55 (4*PFA_2_), as shown in Fig. S10b. Those dynamic evolutions may be ascribed to the changing impedance characteristic of 4*PFA_x_. As to 4*PFA_2_, longer electroless Ag plating time endows it with higher electrical conductivity, thereby bringing an excellent EMI shielding performance (> 20 dB) with a low *T* coefficient and a high *A* and *R* coefficients. Such results indirectly confirm the effectiveness of electroless Ag plating time on modulating the impedance characteristic of 4*PFA_x_.

As a consequence, 4*PFA_x_ can be further assembled with PA_1.5_ to investigate the effect of construction of impedance matching structure on EMI shielding performance of sample. As shown in Fig. 3g, EMI SE_T_ of 4*PFA_x_/PA_1.5_ shows a gradual tendency to increase from 74.1 dB (4*PFA_0_/PA_1.5_) to 77 dB (4*PFA_1_/PA_1.5_), and then to 91.1 dB (4*PFA_2_/PA_1.5_), due to the improved EMI SE performance of 4*PFA_x_ (Fig. S10). More importantly, PFA_x_/PA_1.5_ exhibits ultralow SE_R_ (< 3 dB) in X-band. As displayed in Fig. 3g, 4*PFA_1_/PA_1.5_ possesses the lowest SE_R_ of 0.4 dB, compared to that of 4*PFA_0_/PA_1.5_ (1.7 dB) and 4*PFA_2_/PA_1.5_ (2.8 dB). To analyze the mechanism of EMI shielding performance for 4*PFA_x_/PA_1.5_, the corresponding *A* and *R* coefficients are also calculated and shown in Figs. 3h and S11. It can be obviously seen that *R* coefficient exhibits a tendency to firstly decreases, and then to increase with the increasing Ag content in PFA_x_. Such results confirm the effectiveness of impedance matching structure on low-reflectivity EMI shielding performance. For 4*PFA_0_/PA_1.5_, 4*PFA_0_ possesses a high *T* coefficient due to the low Ag nanoparticle content (Fig. S10d). Hence, EMW can easily penetrate 4*PFA_0_, and sharply be reflected at the surface of PA_1.5_. Furthermore, EMW is rarely dissipated within 4*PFA_0_, resulting in a high *R* and low *A* coefficient for 4*PFA_0_/PA_1.5_. As to 4*PFA_2_/PA_1.5_, the higher Ag nanoparticle content endows 4*PFA_2_ with a high *R* coefficient as shown in Fig. S10c. In this case, most of EMW will be reflected at the surface of 4*PFA_2_, due to the formation of impedance mismatch between air and 4*PFA_2_. Only a few of EMW can enter 4*PFA_2_ to be attenuated. Therefore, a high *R* coefficient of ~ 0.4 can be obtained for 4*PFA_2_/PA_1.5_. In contrast, 4*PFA_1_ with moderate *R* coefficient (~ 0.2) can effectively induce most of EMW enter 4*PFA_1_ and be dissipated as much as possible, owning to the formation of rational impedance matching between air, 4*PFA_1_, and PA_1.5_. Meanwhile, similar tendency arises in the Reflectivity of 4*PFA_x_/PA_1.5_ (Fig. 3i). 4*PFA_1_/PA_1.5_ exhibits the lowest reflectivity, which indicates that the least EMW reflection arises on the surface of 4*PFA_1_/PA_1.5_, compared to the 4*PFA_0_/PA_1.5_ and 4*PFA_2_/PA_1.5_. Also, it implies that the 4*PFA_1_/PA_1.5_ possesses an excellent impedance matching performance. Based on the above, it once again confirms the effectiveness of impedance matching structure on electromagnetic dissipation performance, inducing more EMW to enter the composite and be dissipated as much as possible. Additionally, EMI shielding performance of PA_y_ is analyzed by changing the plating times. As displayed in Fig. S11, the corresponding EMI SE of PA_y_ presents a progressive tendency to increase from 53.1 (1 h) to 67.5 dB (1.5 h), and then to 77.1 dB (2 h). Furthermore, PA_y_ is test by assembling an impedance matching structure (4*PFA_1_/PA_y_) with 4*PFA_1_. As shown in Fig. S13a, the corresponding EMI SE of 4*PFA_1_/PA_y_ exhibits a gradual tendency to increase from 61.3 to 79.1 dB. However, the corresponding *A* coefficient of 4*PFA_1_/PA_y_ demonstrates a minor difference, which firstly increases and then deceases (Fig. S13b). It once again confirms the effectiveness and difference of impedance matching structure on low-reflectivity EMI shielding performance.

**Fig. 4:**
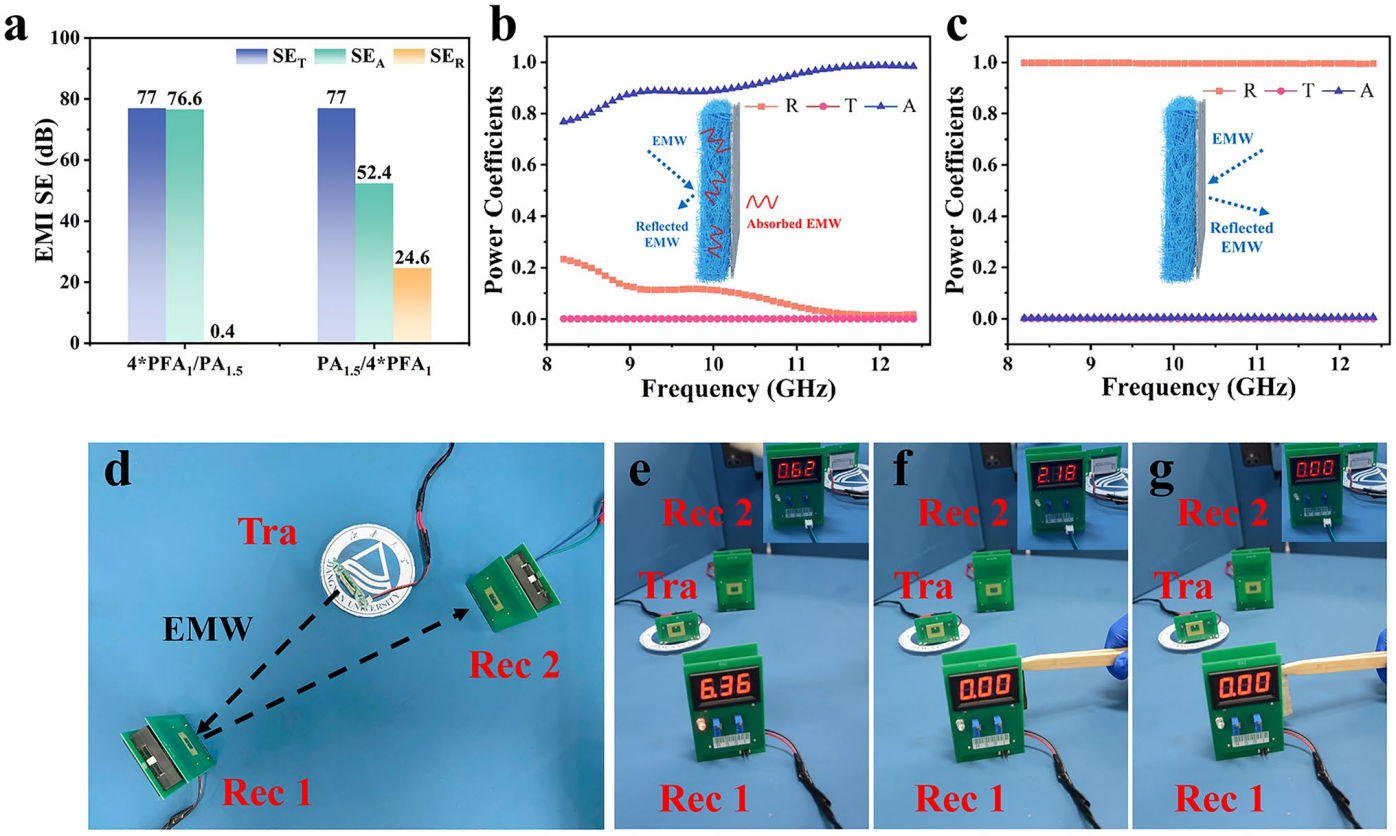
**a** EMI SE of 4*PFA_1_/PA_1.5_ and PA_1.5_/4*PFA_1_. Power coefficients of **b** 4*PFA_1_/PA_1.5_ and **c** PA_1.5_/4*PFA_1_. **d** Demonstration of EMI shielding system; the changes of EMI shielding system inserted with **e** nothing, **f** PA_1.5_/4*PFA_1_, and **g** 4*PFA_1_/PA_1.5_

In order to further clarify the contribution of the impedance matching structure, EMI shielding performances are investigated by controlling the incident direction of EMW. As revealed in Figs. 4a and S14a, SE_T_ exhibits a similar value of 77 dB, when the incident direction of EMW is set from 4*PFA_1_ to PA_1.5_ (4*PFA_1_/PA_1.5_) or from PA_1.5_ to 4*PFA_1_ (PA_1.5_/4*PFA_1_). However, a distinct difference arises in SE_R_ and SE_A_. As shown in Fig. 4a and Fig. S14b, c, 4*PFA_1_/PA_1.5_ exhibits high SE_A_ (~ 76.7 dB) and low SE_R_ (~ 0.4 dB). Conversely, PA_1.5_/4*PFA_1_ demonstrates lower SE_A_ (~ 52.4 dB) and higher SE_R_ (~ 24.6 dB). Accordingly, the power coefficient of 4*PFA_1_/PA_1.5_ and PA_1.5_/4*PFA_1_ can be also calculated to explain the difference in EMI shielding mechanisms. As shown in Fig. 4b, c, the ultralow *R* coefficient (< 0.09) for 4*PFA_1_/PA_1.5_ contrasts sharply with the ultrahigh *R* coefficient (~ 1) for PA_1.5_/4*PFA_1_. It once again confirms the effectiveness of impedance matching structure on low-reflectivity EMI shielding performance.

To clearly illustrate the different EMI shielding performances of 4*PFA_1_/PA_1.5_ and PA_1.5_/4*PFA_1_, a transmitter (Tra) and two receivers (Rec 1 and Rec 2) capable of transmitting 1.0 W EMW at 10.5 GHz by antenna are fabricated as demonstrated in Fig. 4d. The number displayed on the receiver is proportional to the power of received EMW. More importantly, after being received by Rec 1, some EMW are reflected on the surface of Rec 1, and will be received by Rec 2. As a result, Rec 1 and Rec 2 exhibit the different numbers on the digital display. As shown in Fig. 4e, when the receiver accepts the wireless signal without any interference, Rec 1 shows the higher number (6.36), due to its directly received EMW generated from Tra. Meanwhile, a part of received EMW is reflected on the surface of Rec 1, due to the existence of metal components within Rec 1. As a result, Rec 2 receives a few of EMW from Rec 1, and exhibits smaller numbers (0.62). Based on the above configurations, PA_1.5_/4*PFA_1_ is inserted between Rec 1 and Rec 2. Moreover, 4*PFA_1_ faces to Rec 1, while PA_1.5_ faces to Rec 2. The number of Rec 1 and Rec 2 rapidly turn to 0 and 2.18 (Fig. 4f and Movie S1), respectively. In contrast, the number of Rec 1 and Rec 2 sharply undergoes zero when 4*PFA_1_/PA_1.5_ is inserted between Rec 1 and Rec 2 (Fig. 4g and Movie S2). It once again indicates that 4*PFA_1_/PA_1.5_ nonwoven fabric effectively inhibits the reflectivity of EMW and possesses high EMI shielding performance.

### Infrared Stealth Performance of Composite Nonwoven Fabrics

Thermal radiation continuously produces for all objects with temperature above absolute zero. Especially, high-temperature thermal radiation generated from equipment operation can cause significant radiation, which can be easily detected by infrared detector. Therefore, high-performance infrared stealth materials should be designed and fabricated by inhibiting those infrared radiations of objects. To characterize the infrared stealth performance of those nonwoven fabrics, the IR reflectivity in mid-infrared wavelengths is measured by FTIR spectrometer using an integrating sphere. From Fig. S15, IR reflectivity of PA_x_ exhibits a tendency to increase from ~ 40% to ~ 60%, and then decrease to 55% with the prolonging Ag plating time from 1 to 1.5 and 2 h. It can be attributed to the changing Ag content and roughness on the surface of PA_x_ fiber. Fewer Ag nanoparticles plated at a short time of 1h cannot completely cover the surface of PI fibers. Those exposed area of PI uncovered by Ag nanoparticles exhibits a low IR reflectivity characteristic, thereby endowing PA_1_ with a lower IR reflectivity. Furthermore, excessed Ag nanoparticles plated at a long time of 2 h leads to the generation of aggregation on the surface of PI fibers, and an increase in roughness. It induces the formation of scattered reflection of IR wave, resulting in a decrease in IR reflectivity for PA_2_.

**Fig. 5:**
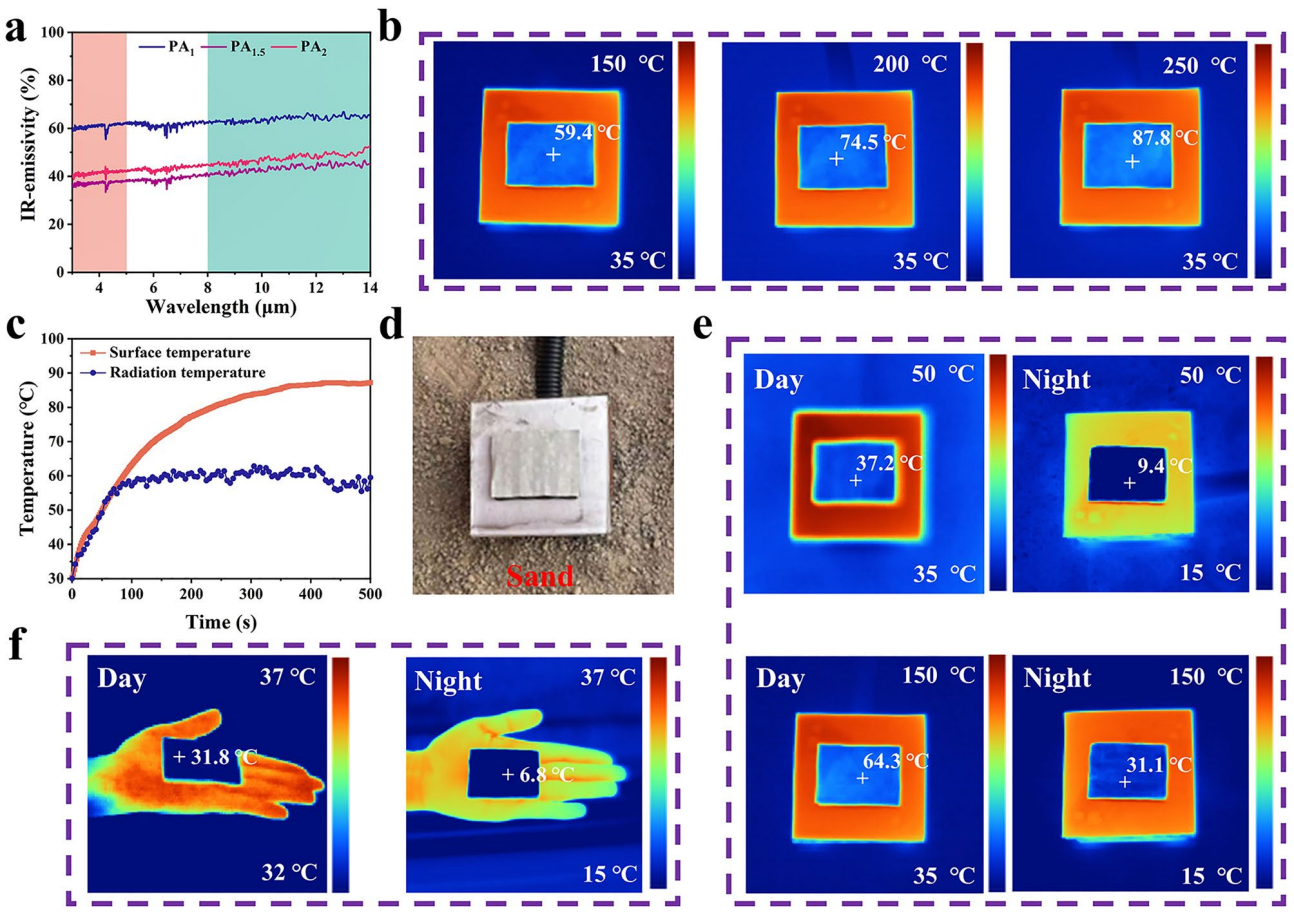
**a** IR emissivity curves of PA_x_. **b** IR images of 4*PFA_1_/PA_1.5_ on the thermal stages of 150, 200, and 250 °C. **c** Surface and radiation temperature curves of 4*PFA_1_/PA_1.5_ on the thermal stages of 150 °C. **d** Schematic diagram under simulated desert environment. **e** IR images of 4*PFA_1_/PA_1.5_ in the simulated desert environment under different thermal stage temperatures. **f** IR images of 4*PFA_1_/PA_1.5_ on palm

According to Eq. 9, the corresponding IR emissivity of PA_x_ can be calculated and recorded in Fig. 5a. The obtained IR emissivity shows an opposite tendency with IR reflectivity. Very importantly, PA_1.5_ possesses the lowest IR emissivity of 0.36 and 0.43 within 3–5 and 8–14 μm, respectively. Additionally, the formation of fluffy 3D space structure between fibers endows 4*PFA_1_ with an excellent thermal insulation capability. To observe the infrared stealth performance, 4*PFA_1_/PA_1.5_ is placed on a thermal stage with different temperature (50–250 °C) and tested by infrared thermographer (FLUKE Ti400 +). As shown in Figs. S16 and 5b, 4*PFA_1_/PA_1.5_ displays an outstanding infrared stealth performance. The area covered by 4*PFA_1_/PA_1.5_ can be well hidden in infrared image, which is close to deep-blue color of the background. It is worth noting that the corresponding infrared stealth performance can be maintained at the thermal stage with a higher temperature of 250 °C. It indicates that 4*PFA_1_/PA_1.5_ can be employed to inhibit the thermal radiation derived from high-temperature source. To further analyze the infrared stealth performance, the surface and radiation temperature of the samples are measured and recorded on a 150 °C thermal stage. As shown in Fig. 5c, the surface temperature of 4*PFA_1_/PA_1.5_ measured by a thermocouple reaches 81.2 °C after 500 s. The difference in temperature between the surface of sample and thermal stage can be up to 62.8 °C. It can be attributed to the excellent thermal insulation capacity of nonwoven fabric. As a consequence, a lower radiation temperature of 60 °C can be easily observed by infrared thermographer after 500 s. Such outstanding high-temperature resistant infrared stealth performance can be due to the synergistic effect of lowest IR emissivity and thermal insulation of 4*PFA_1_/PA_1.5_. The prominent high-temperature resistant characteristic can be ascribed to the strong bonding force originated from in-situ growth of magnetic particles and "self-activated" electroless Ag plating process.

Notably, the application scenarios of infrared stealth materials may be transformed in practice, such as day and night. Very importantly, the infrared radiation of high-temperature thermal source and background in different scenarios present large differences. Therefore, infrared stealth materials are required to possess better environmental compatibility to adapt to changes in the background. Specifically, the large day/night temperature differences in desert environments are highly probable to contribute to infrared exposure. Accordingly, 4*PFA_1_/PA_1.5_ is also placed in a simulated desert environment to monitor the infrared stealth performance during day and night. As demonstrated in Fig. 5e, excellent infrared stealth performance can be readily achieved for different thermal sources (50 and 150 °C) at day and/or night. In addition, 4*PFA_1_/PA_1.5_ exhibits an excellent thermal camouflage for the human body (palm) as shown in Fig. 5f. During the day and night, 4*PFA_1_/PA_1.5_ displays a darker blue color and lower temperature compared to its surroundings.

### Compatible EMI Shielding/Infrared Stealth Performance of Composite Nonwoven Fabrics

**Fig. 6:**
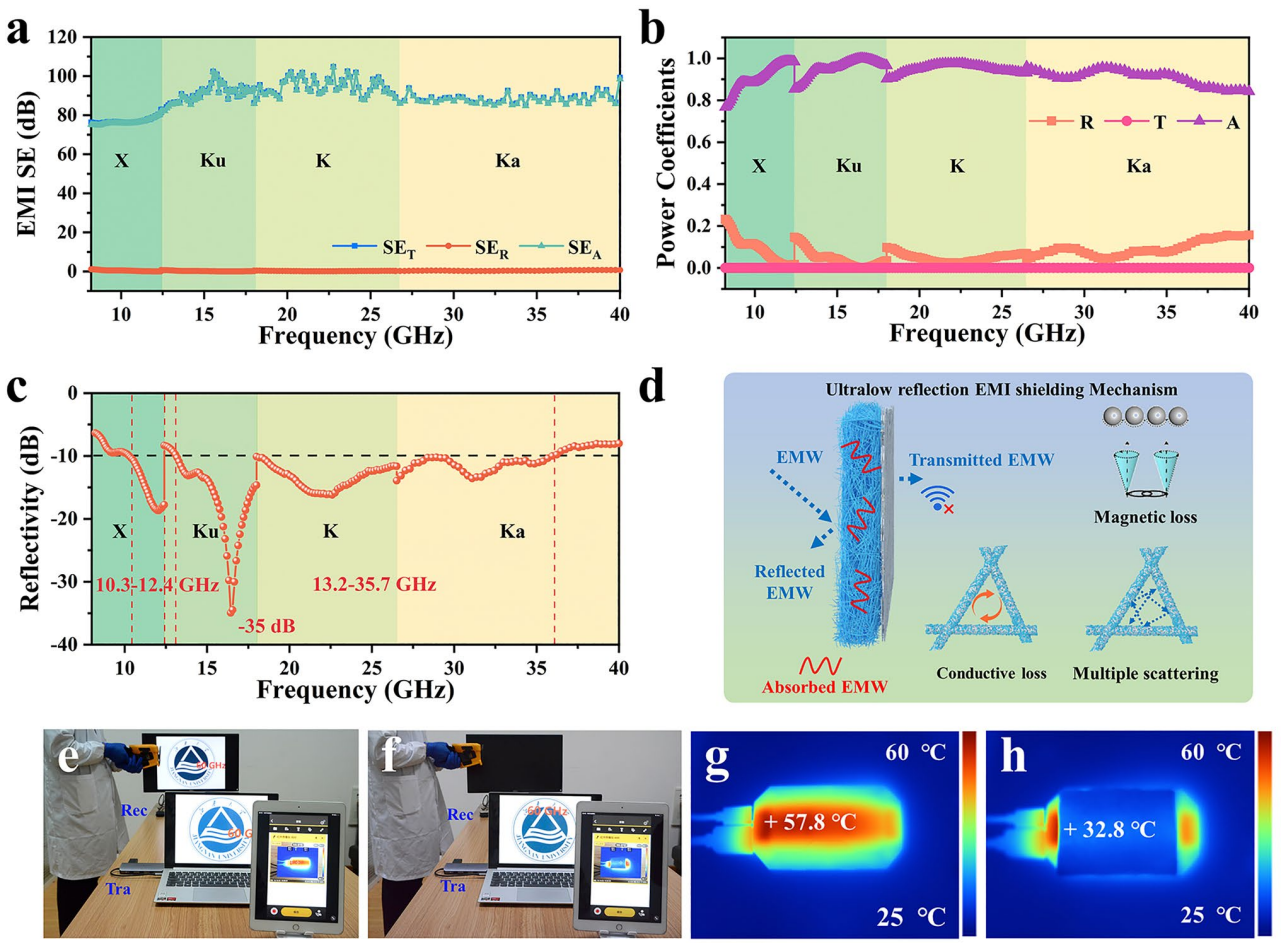
**a** EMI SE, **b** power coefficients, and **c** reflectivity of 4*PFA_1_/PA_1.5_ at 8.2–40 GHz. **d** Low-reflectivity EMI shielding mechanism diagram of 4*PFA_1_/PA_1.5_. Demonstration of the compatible EMI shielding and infrared stealth performance of 4*PFA_1_/PA_1.5_ in the state of **e** original and **f** the receiver covered by 4*PFA_1_/PA_1.5_. IR images of **g** original and **h** receiver covered by 4*PFA_1_/PA_1.5_

With the iterative upgrading of communications technologies, the working frequency of electrical equipment gradually shifts to the higher frequency range. As shown in Fig. 6a, the EMI SE of 4*PFA_1_/PA_1.5_ is higher than 76 dB in the typical frequency range including X-band (8.2–12.4 GHz), Ku-band (12.4–18 GHz), K-band (18–26.5 GHz), and Ka-band (26.5–40 GHz). It further verifies the excellent broadband EMI shielding performance and application potential of 4*PFA_1_/PA_1.5_. More importantly, the rational construction of impedance matching in 4*PFA_1_/PA_1.5_ endows it with an ultrahigh *A* (> 0.76) and ultralow *R* (< 0.24) in 8.2–40 GHz (Fig. 6b). Meantime, as shown in Fig. 6c, 4*PFA_1_/PA_1.5_ nonwoven fabric exhibits a broadband microwave absorption performance (< -10 dB) in the range of 10.3–12.4 GHz and 13.2–35.7 GHz. It suggests that more than 90% of incident EMW are absorbed, and less than 10% of incident EMW are reflected. It indicates that the composite possesses an excellent microwave absorption performance. Such result can be once again ascribed to the synergistic effect of impedance matching and various attenuation characteristic of 4*PFA_1_/PA_1.5_, such as multiple scanting, conductive loss, dielectric loss, and magnetic loss (Fig. 6d). As a comparison, recently reported low-reflectivity EMI shielding materials and their performance are listed in Table S1 [30, 50–58]. As summarized in Table S1, 4*PFA_1_/PA_1.5_ presents a significant advantage over other materials. For the compact film, an outstanding EMI SE can be easily achieved at a small thickness, whereas their high electrical conductivity severely hinders the entrance of incident EMW. For example, Guo et al. successfully fabricates an alternating multilayered film containing a CoFe_2_O_4_@MXene/cellulose nanofiber (CNF) layers and silver nanowires (AgNWs)/CNF layers [58]. A higher EMI SE performance of 87.8 dB can be reached at a low thickness of 0.1 mm. However, its high electrical conductivity (793.4 S cm^−1^) results in a high *R* coefficient of 0.72. As to the foam or aerogel, low-reflectivity EMI shielding performance can be easily achieved, but a larger thickness is necessary. For instance, He et al. prepares silica/carbon nanotubes/polyimide-silver nanowires/cellulose nanofibers based on the principle of split-conductive module design [57]. Under the synergistic effect of various EMI shielding mechanisms, an excellent EMI SE of 117 dB can be obtained for the foam. The corresponding *R* coefficient reaches a low value of 0.005 at a big thickness of 10 mm. In practice, the huge thickness significantly reduces the convenience of materials. In this work, the average EMI SE of 4*PFA_1_/PA_1.5_ with a density of 0.17 g cm^−3^ can be up to ~ 87 dB in 8.2–40 GHz at a smaller thickness of 2.46 mm. Importantly, the corresponding *R* coefficient reaches a low value of 0.07. Such excellent low-reflectivity EMI shielding performance makes it possible for 4*PFA_1_/PA_1.5_ to become an efficient EMI shielding materials with a significant competitive advantage.

**Fig. 7:**
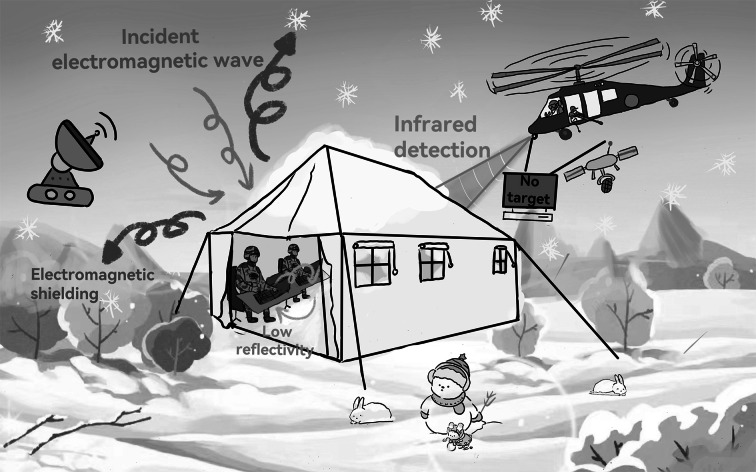
The possible application scenario of 4*PFA_1_/PA_1.5_ with compatible low-reflectivity EMI shielding and infrared stealth performance

To visually illustrate the compatible EMI shielding and infrared stealth performance of 4*PFA_1_/PA_1.5_, a demonstration is presented at a frequency of 60 GHz. As demonstrated in Fig. 6e–h and Movie S3, a display is connected to the notebook by a wireless projector (Tra) and receiver (Rec) in 60 GHz. Under normal condition, the display can show the same animation with notebook in real time by the wireless Rec (Fig. 6e). Meantime, to detect the thermal radiation of Rec in real time, the infrared thermographer is also connected to iPad by WiFi. Normally, the strong thermal radiation of Rec can be easily detected by infrared thermographer as shown in Fig. 6g. However, when 4*PFA_1_/PA_1.5_ covers Rec, the animation of display immediately stop (Fig. 6f). Moreover, those strong thermal radiation of Rec can be instantly inhibited (Fig. 6h). Subsequently, as 4*PFA_1_/PA_1.5_ is removed from Rec, the animation recovers at once, while the corresponding strong thermal radiation arises again. Based on the above, it can be clearly concluded that 4*PFA_1_/PA_1.5_ possesses an excellent compatible EMI shielding and infrared stealth performance. As a consequence, excellent low-reflectivity EMI shielding performance and infrared stealth performance make it promising for 4*PFA_1_/PA_1.5_ applied in military tents (Fig. 7). In detail, the EMW emitted by the electrical equipment in military tents generates low electromagnetic reflection and high electromagnetic absorption on nonwoven fabric. It can effectively avoid EMW leakage from military tents and reduce potential secondary pollution/interference to electronic equipment and human in military tents. Importantly, electromagnetic interference from enemy can be also shielding by 4*PFA_1_/PA_1.5_ nonwoven fabric. In addition, the thermal conductivity and infrared emissivity of 4*PFA_1_/PA_1.5_ nonwoven fabric are low, thereby endowing the military tents with an excellent infrared stealth performance. In particular, the cold environment increases the possibility of infrared exposure of military equipment/humans. Excellent infrared stealth performance of 4*PFA_1_/PA_1.5_ can effectively reduce infrared radiation, achieving infrared stealth for military equipment/humans in cold environment.

## Conclusions

In summary, a hierarchical polyimide-based nonwoven fabric for ultralow-reflectivity EMI shielding/high-temperature resistant infrared stealth is successfully prepared by alkali treatment, in-situ growth of magnetic particles and "self-activated" electroless Ag plating process. Originated from the hydrolyzation of imide rings in PI molecules, the carboxylic active sites can be served as strong bonding anchors for Ag nanoparticles generated by Ag^+^ exchange and chemical reduction, thereby facilitating the formation of "self-activated" Ag-coated PI nonwoven fabric (PA). Additionally, those carboxylic active sites provide strong adhesion for in-situ growth of Fe_3_O_4_ nanoparticles through complexation with Fe^3+^ and annealing treatment. Meanwhile, DA can be employed to polymerize and increase the quantity of active sites on the surface of Fe_3_O_4_-loaded PI nonwoven fabric. Those residual catechol and amine functional groups in PDA effectively promote the in-situ reduction of Ag^+^ to Ag nanoparticle, accelerating the "self-activated" electroless silver plating process. As a result, the impedance characteristic of Fe_3_O_4_/Ag-loaded PI nonwoven fabrics (PFA) can be easily adjusted. The synergistic fabrication of PFA and PA promotes the rational construction of hierarchical impedance matching in PFA/PA. It induces more EMW to enter the composite and be dissipated as much as possible, endowing it with an ultralow-reflectivity EMI shielding performance (SE_T_ = 77 dB, *R* = 0.09). Moreover, the thermal insulation of fluffy 3D space structure in PFA and IR emissivity of PA originated from Ag plating bring an excellent infrared stealth performance. More importantly, the strong adhesion interaction between Fe_3_O_4_, Ag, and PI fiber allows it resist the thermal stress derived from high-temperature source (250 °C), enhancing the thermal stability in EMI shielding and high-temperature resistant infrared stealth performance. Such excellent compatible ultralow-reflectivity EMI shielding/high-temperature resistant infrared stealth performance makes it possible for PFA/PA to be applied in military tent and/or camouflage.

## Supplementary Information

Below is the link to the electronic supplementary material.

Supplementary file1 (MP4 9612 KB)

Supplementary file2 (MP4 9926 KB)

Supplementary file3 (MP4 33817 KB)

Supplementary file4 (DOCX 10804 KB)
