# Supplementary material for: Hierarchical Polyimide Nonwoven Fabric with Ultralow-Reflectivity Electromagnetic Interference Shielding and High-Temperature Resistant Infrared Stealth Performance
Source: Nanomicro Lett. 2024 Dec 3;17:82. doi: 10.1007/s40820-024-01590-3 (PMC11615167; doi:10.1007/s40820-024-01590-3)
Supplement: Supplementary file 4 — Supplementary file4 (DOCX 10804 KB) [file 40820_2024_1590_MOESM4_ESM.docx]

Supporting Information for

**Hierarchical Polyimide Nonwoven Fabric with Ultralow-Reflectivity Electromagnetic Interference Shielding and High-Temperature Resistant Infrared Stealth Performance**

Xinwei Tang^1^, Yezi Lu^1^, Shuangshuang Li^1^, Mingyang Zhu^1^, Zixuan Wang^1^, Yan Li^2^, Zaiyin Hu^3^, Penglun Zheng^4^, Zicheng Wang^1,^* and Tianxi Liu^1,^*

^1^ The Key Laboratory of Synthetic and Biological Colloids, Ministry of Education, School of Chemical and Material Engineering, International Joint Research Laboratory for Nano Energy Composites, Jiangnan University, Wuxi, Jiangsu 214122, P. R. China

^2^ Jiangsu Ferrotec Semiconductor Technology Co., Ltd. Yancheng, Jiangsu 214000, P. R. China

^3^ Guizhou Aerospace Wujiang Electro-mechanical Equipment Co., Ltd. 563000, No. 20-5, Dalian Road Aerospace Industrial Park, Huichuan District, Zunyi City, Guizhou Province, P. R. China

^4^ Civil Aircraft Fire Science and Safety Engineering Key Laboratory of Sichuan Province, College of Civil Aviation Safety Engineering, Civil Aviation Flight University of China, Guanghan 618307, P. R. China.

*Corresponding authors. E-mail: [wangzc@jiangnan.edu.cn](mailto:wangzc@jiangnan.edu.cn) (Zicheng Wang); [txliu@jiangnan.edu.cn](mailto:txliu@jiangnan.edu.cn) (Tianxi Liu)

Supplementary Figures

**
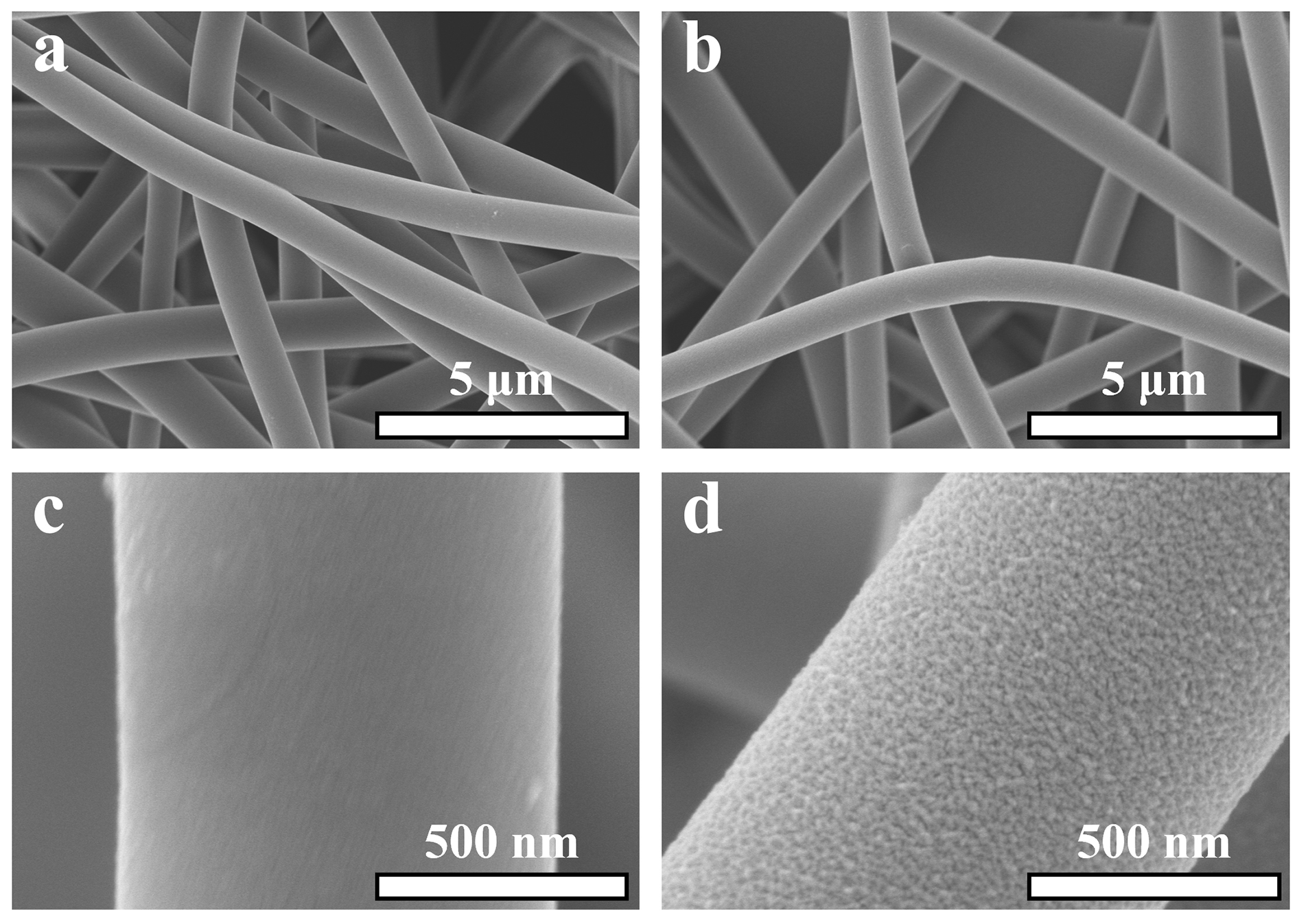
**

**Fig. S1** SEM images of (**a, c**) PI, and (**b, d**) PI-NaOH fiber


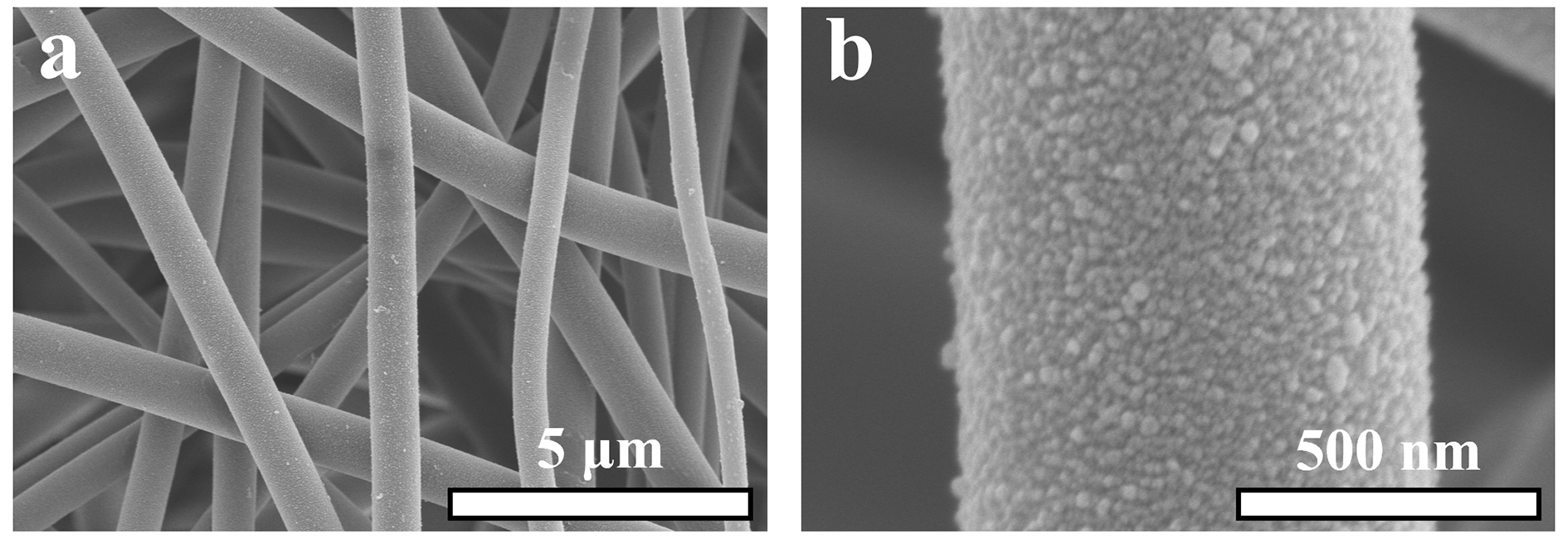


**Fig. S2** (**a, b**) SEM images of PI fiber after activation


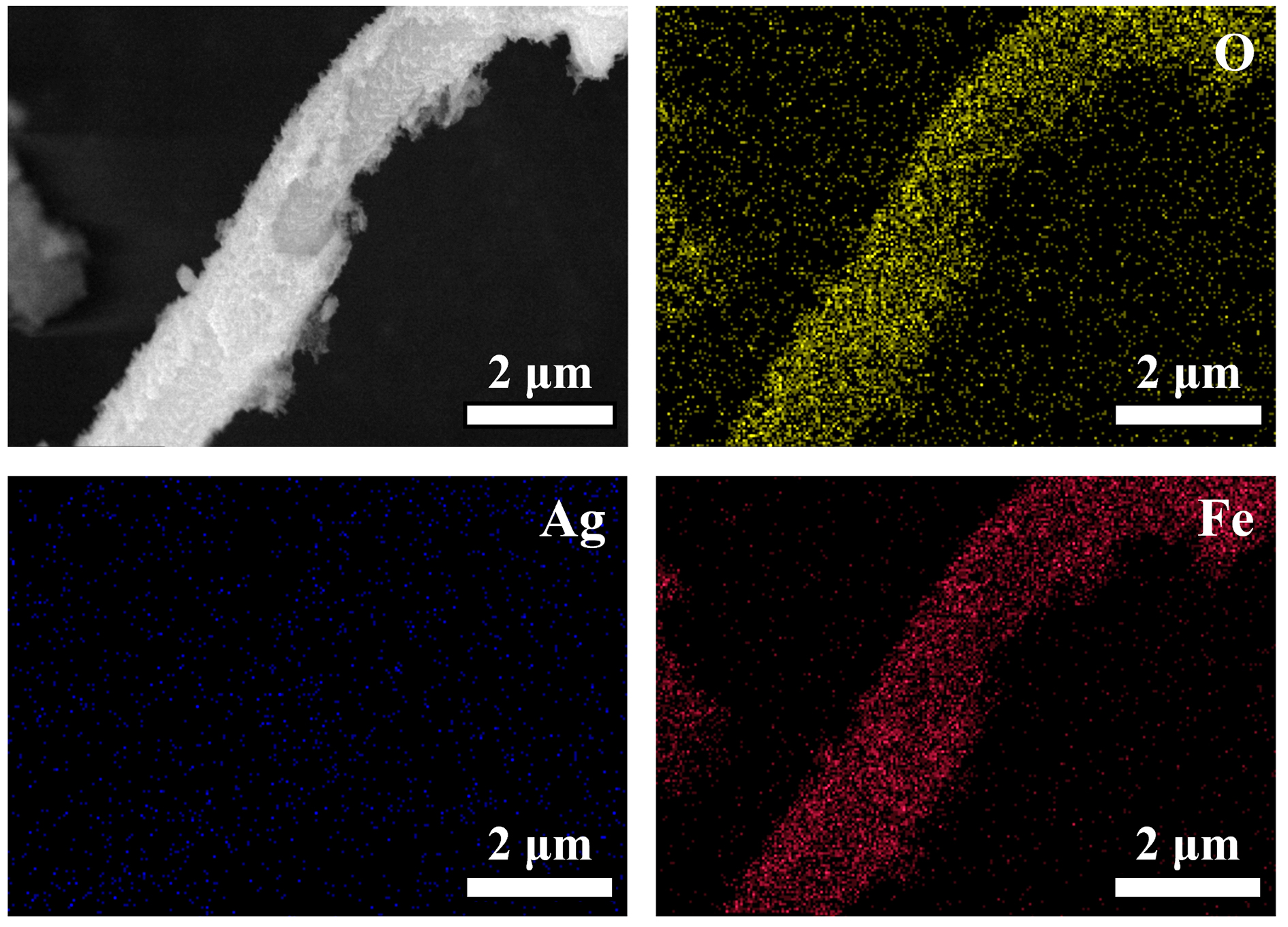


**Fig. S3** EDS mapping images of PFA_0_ fiber


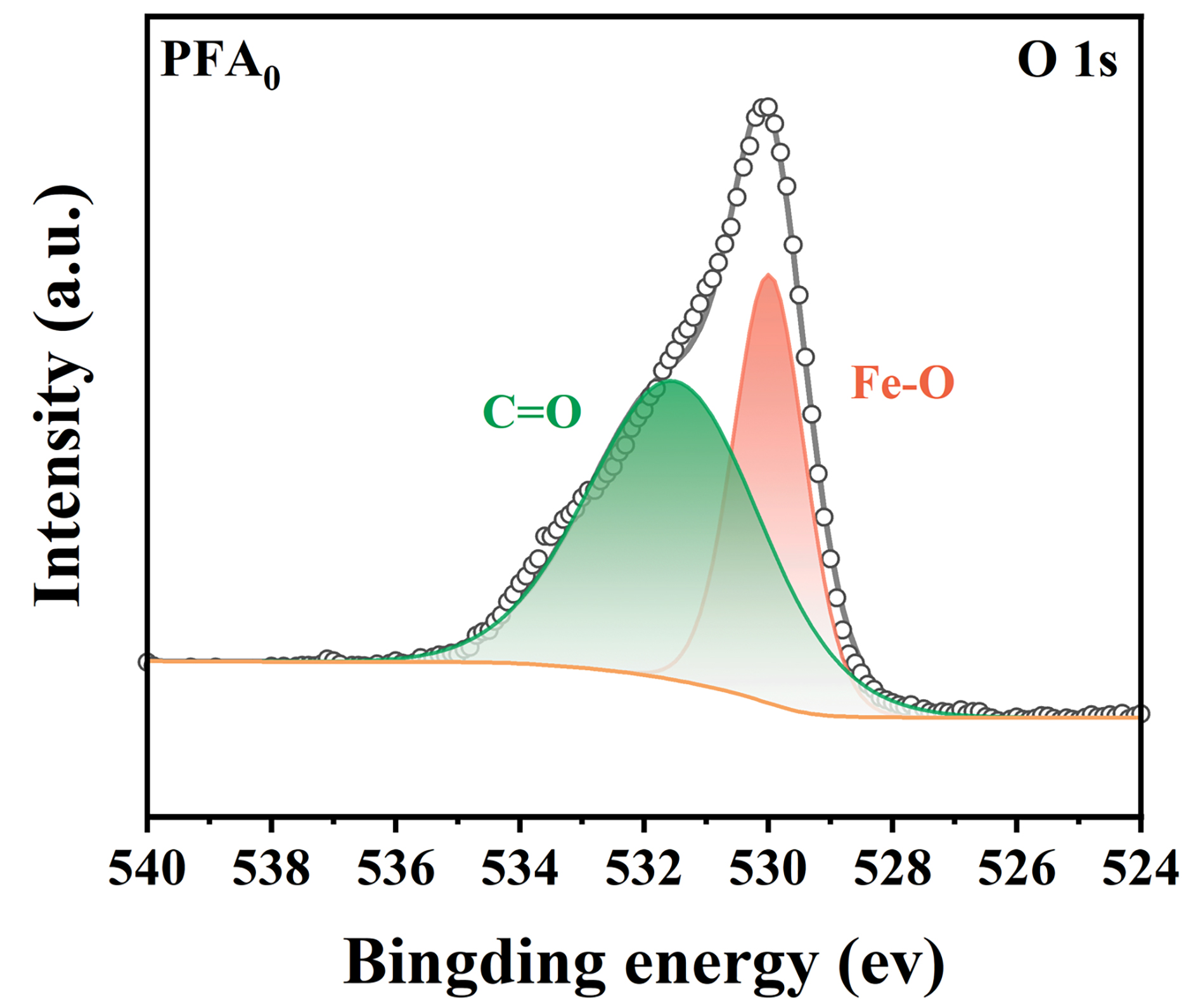


**Fig. S4** High resolution XPS spectra of O 1s for PFA_0_ nonwoven fabric


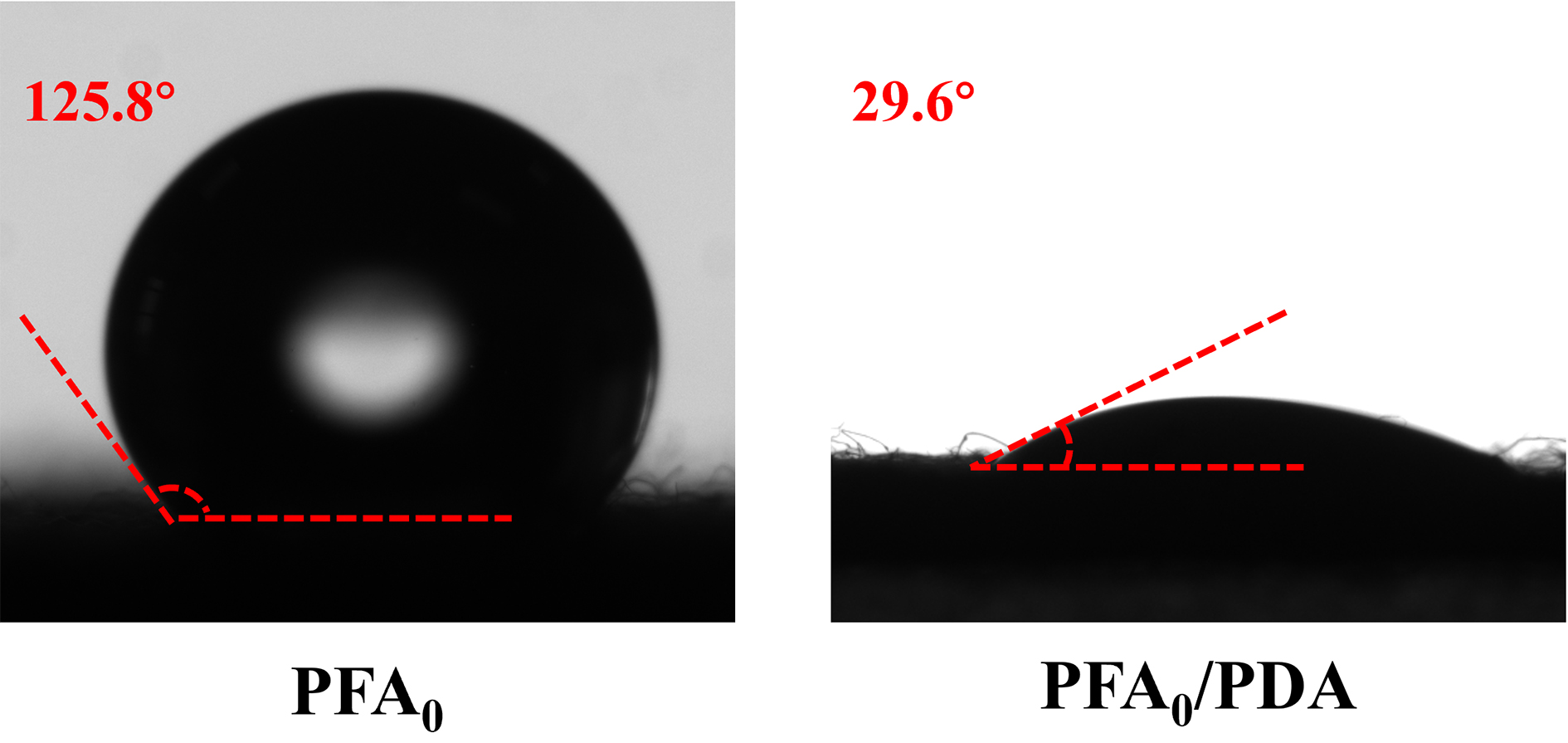


**Fig. S5** Water contact angles of PFA_0_ and PFA_0_/PDA nonwoven fabrics


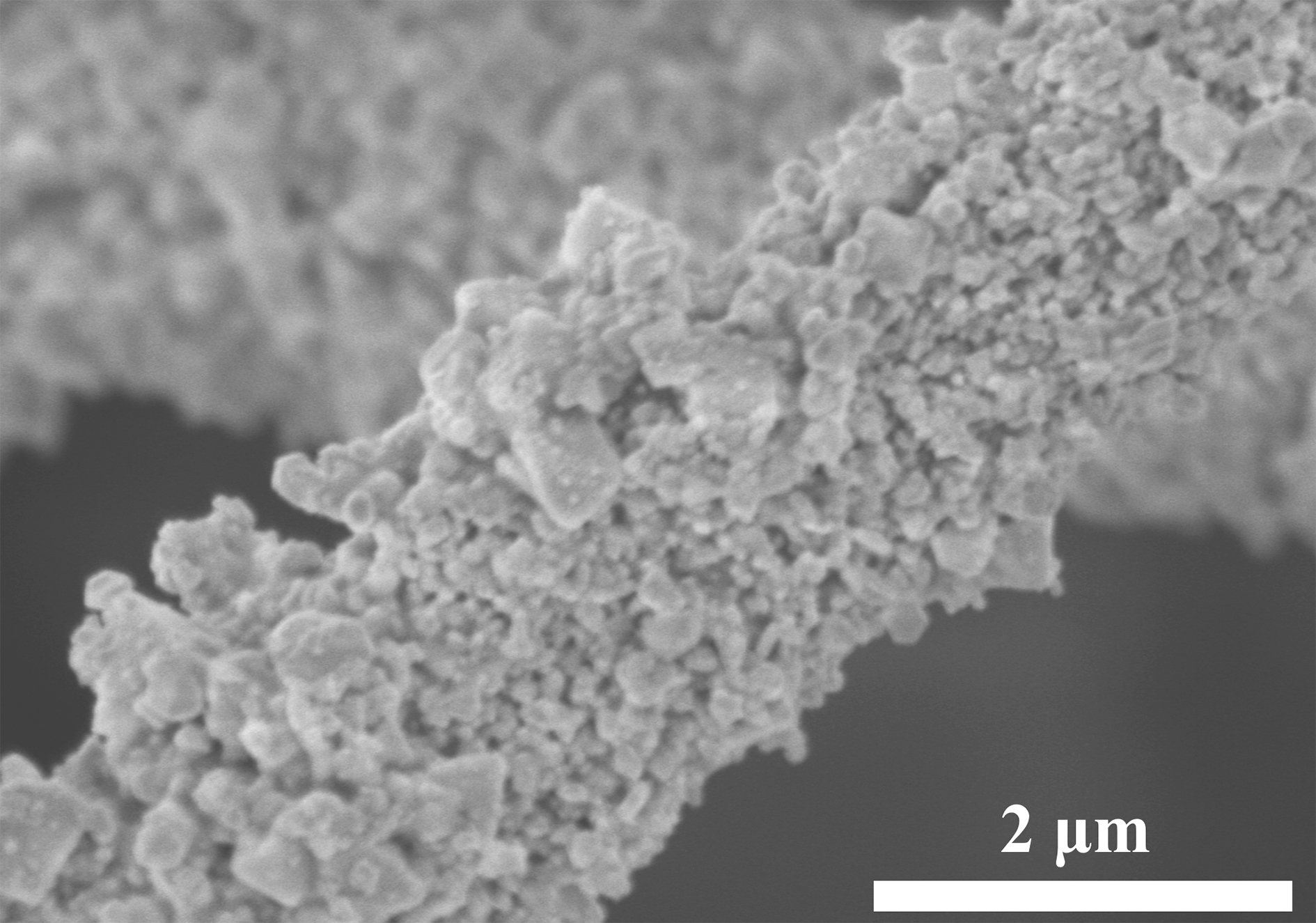


**Fig. S6** SEM images of PFA_1_ nonwoven fabric


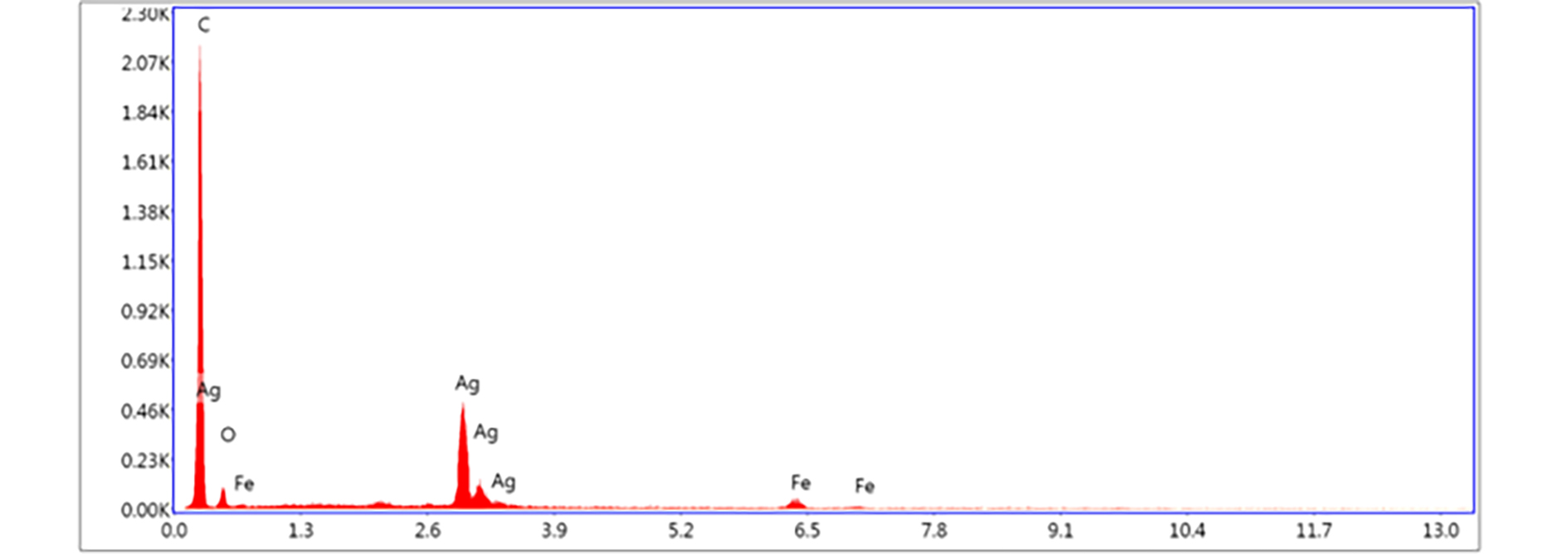


**Fig. S7** EDS spectra of PFA_1_ nonwoven fabric


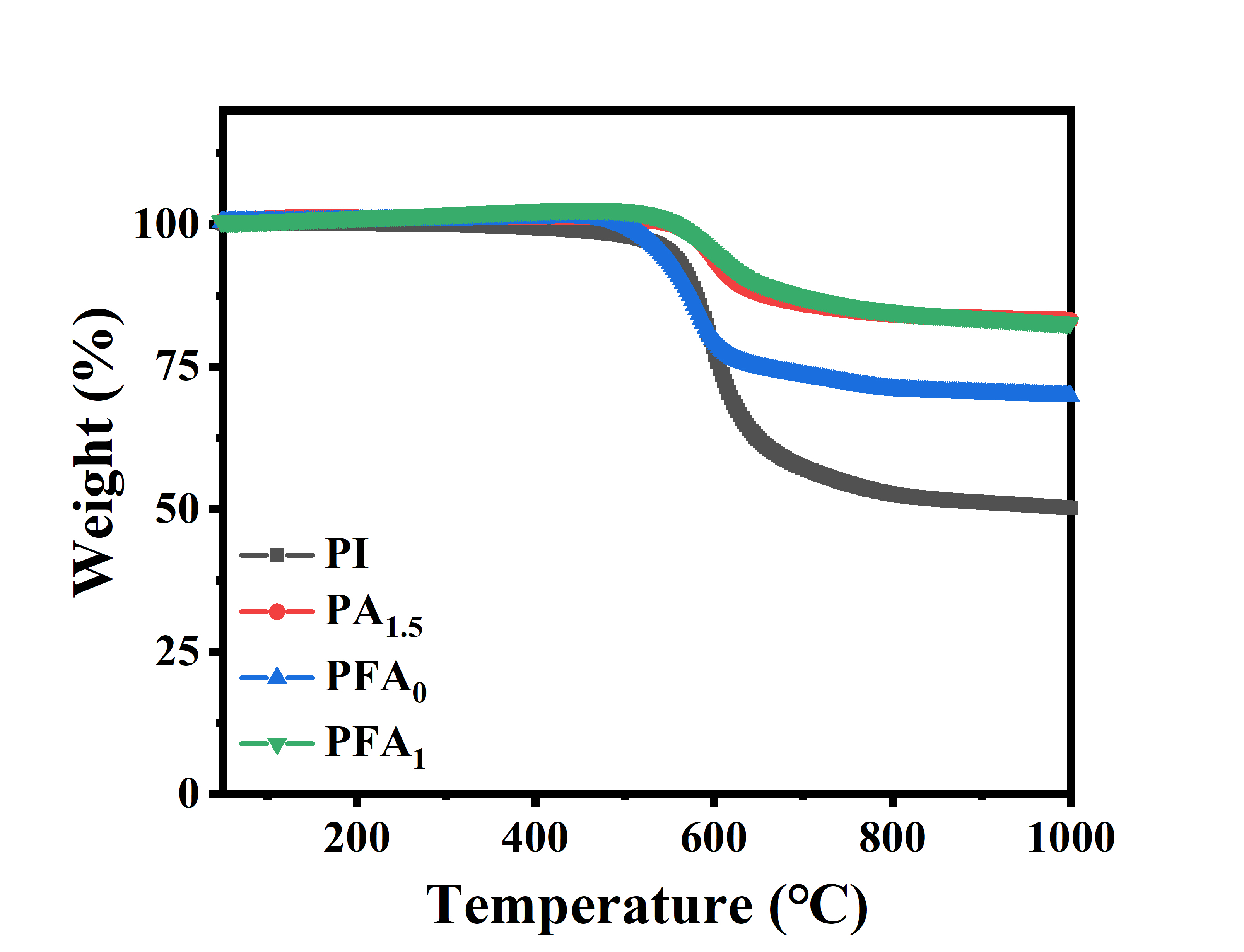


**Fig. S8** TGA curves of PI, PA_1.5_, PFA_0_ and PFA_1_ nonwoven fabrics


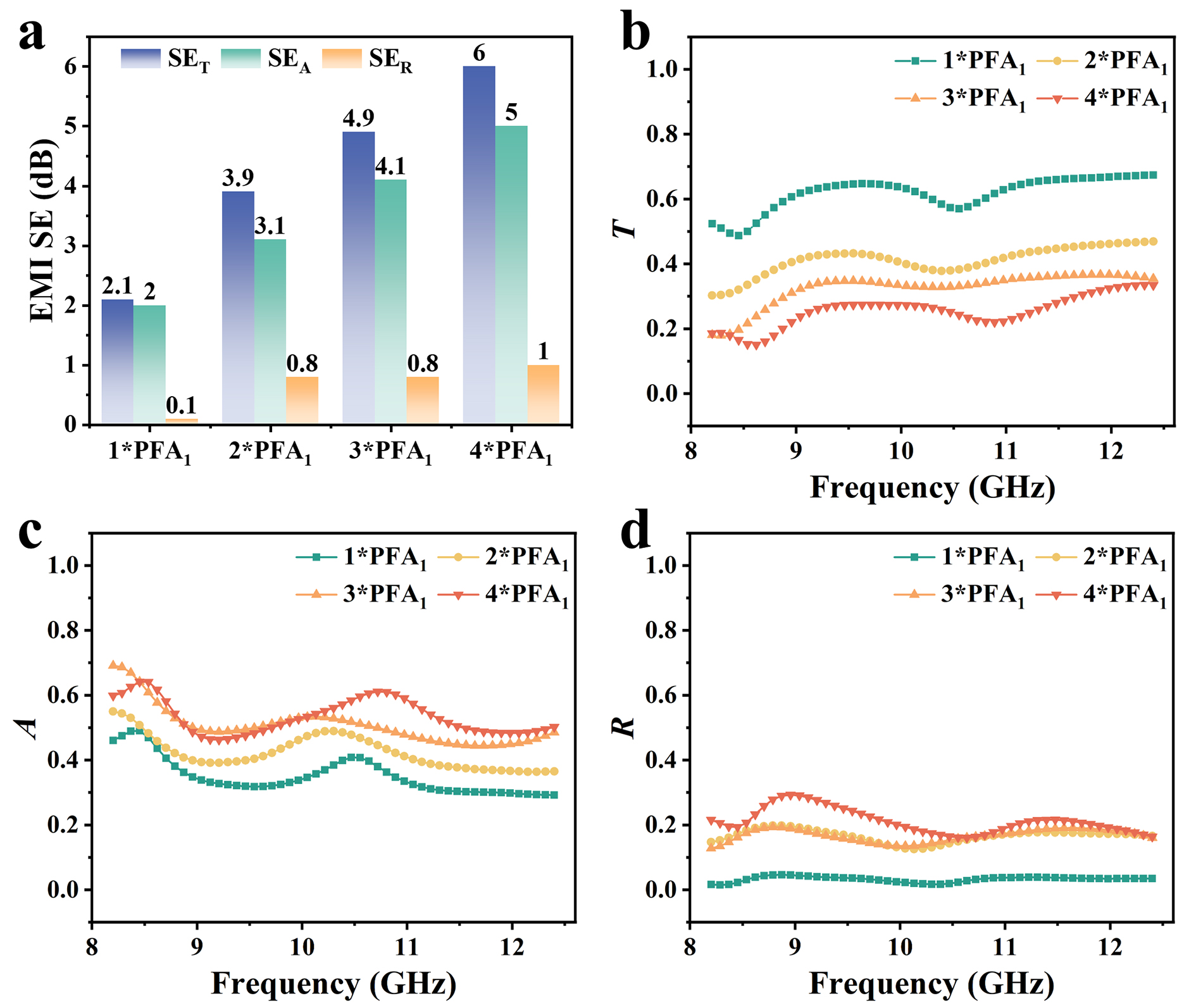


**Fig. S9** (**a**) EMI SE, (**b**) *T*, (**c**) *A* and (**d**) *R* of N*PFA_1_


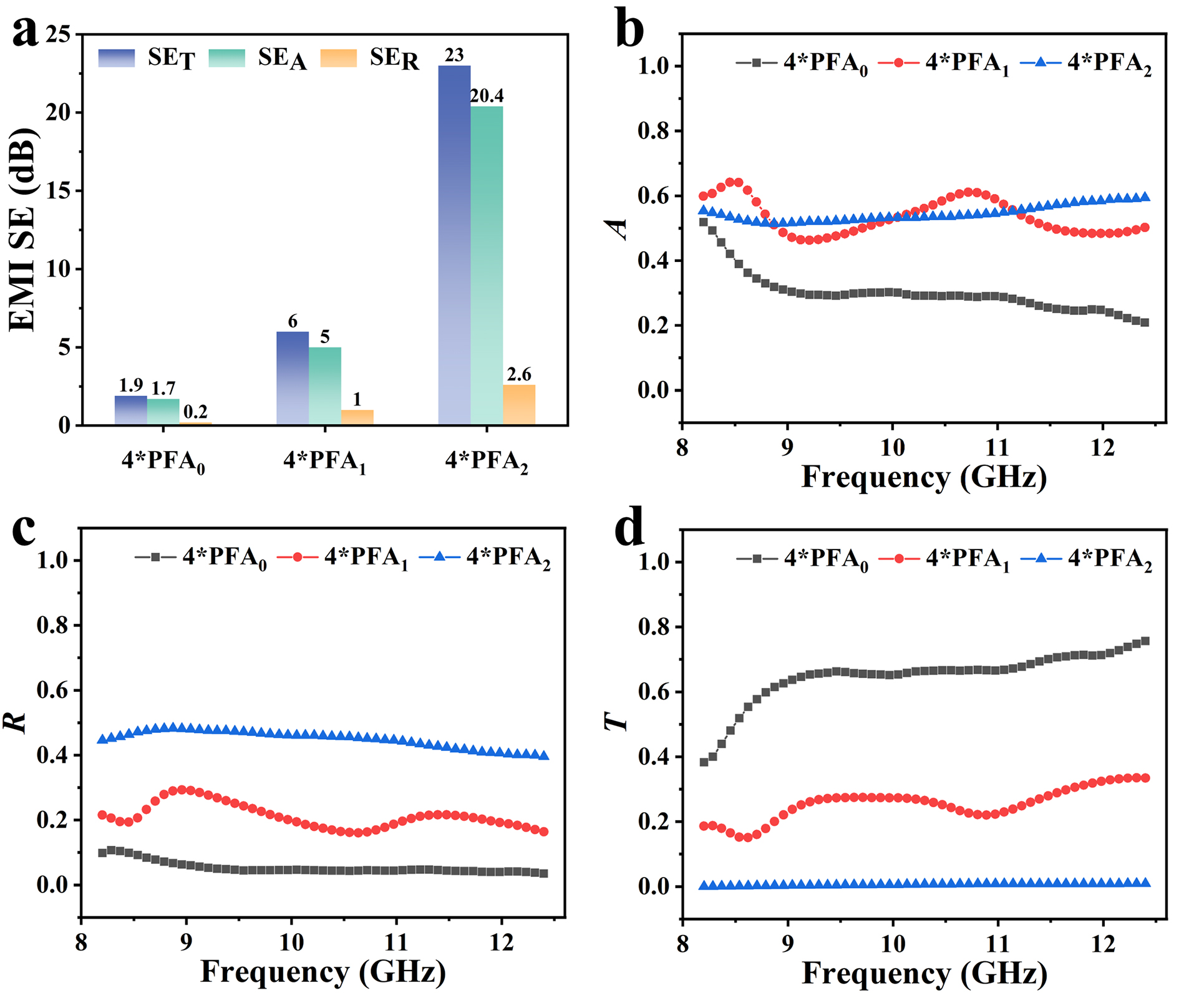


**Fig. S10** (**a**) EMI SE, (**b**) *A*, (**c**) *R* and (**d**) *T* of 4*PFA_x_


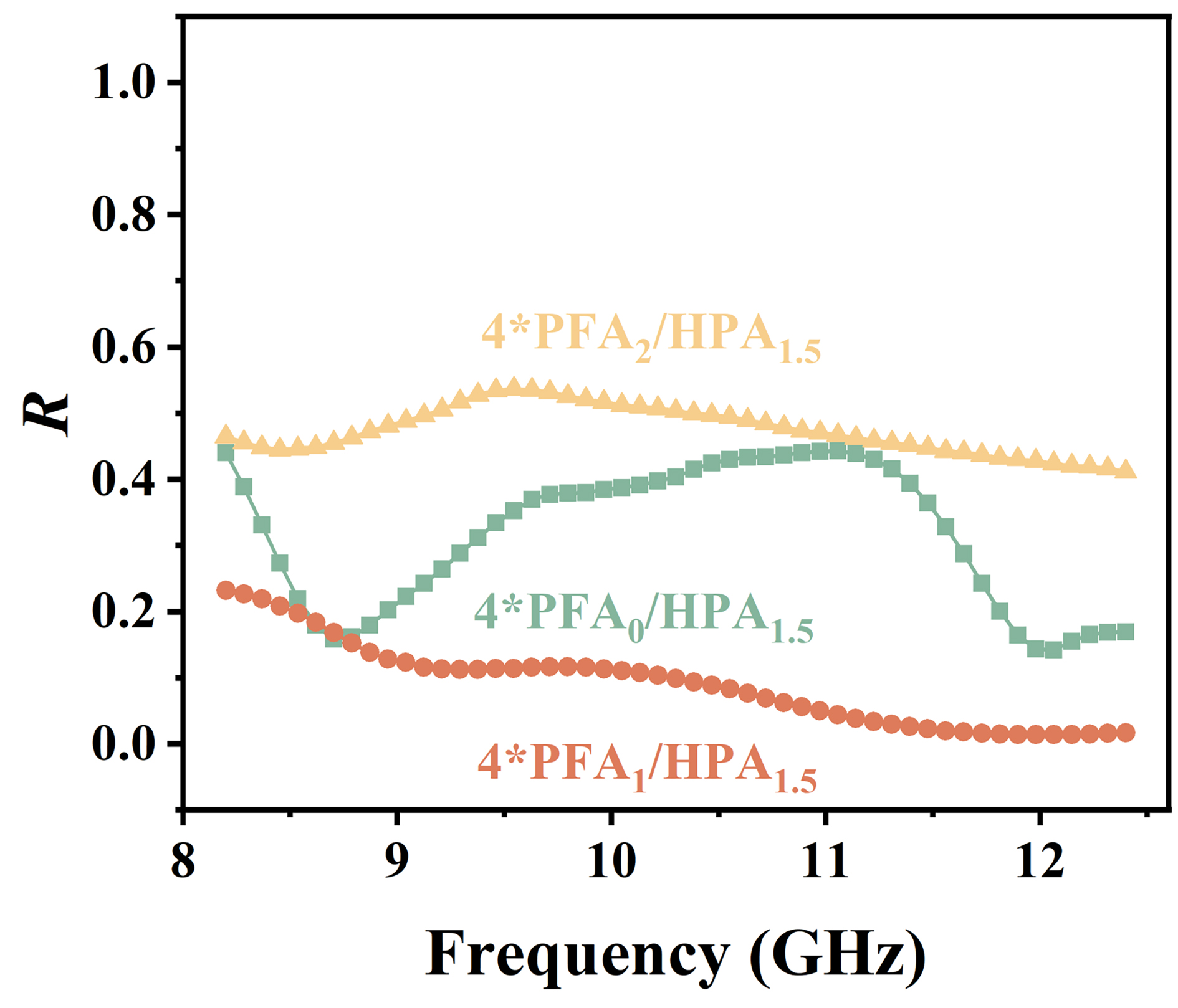


**Fig. S11** *R* of 4*PFA_x_/PA_1.5_

**
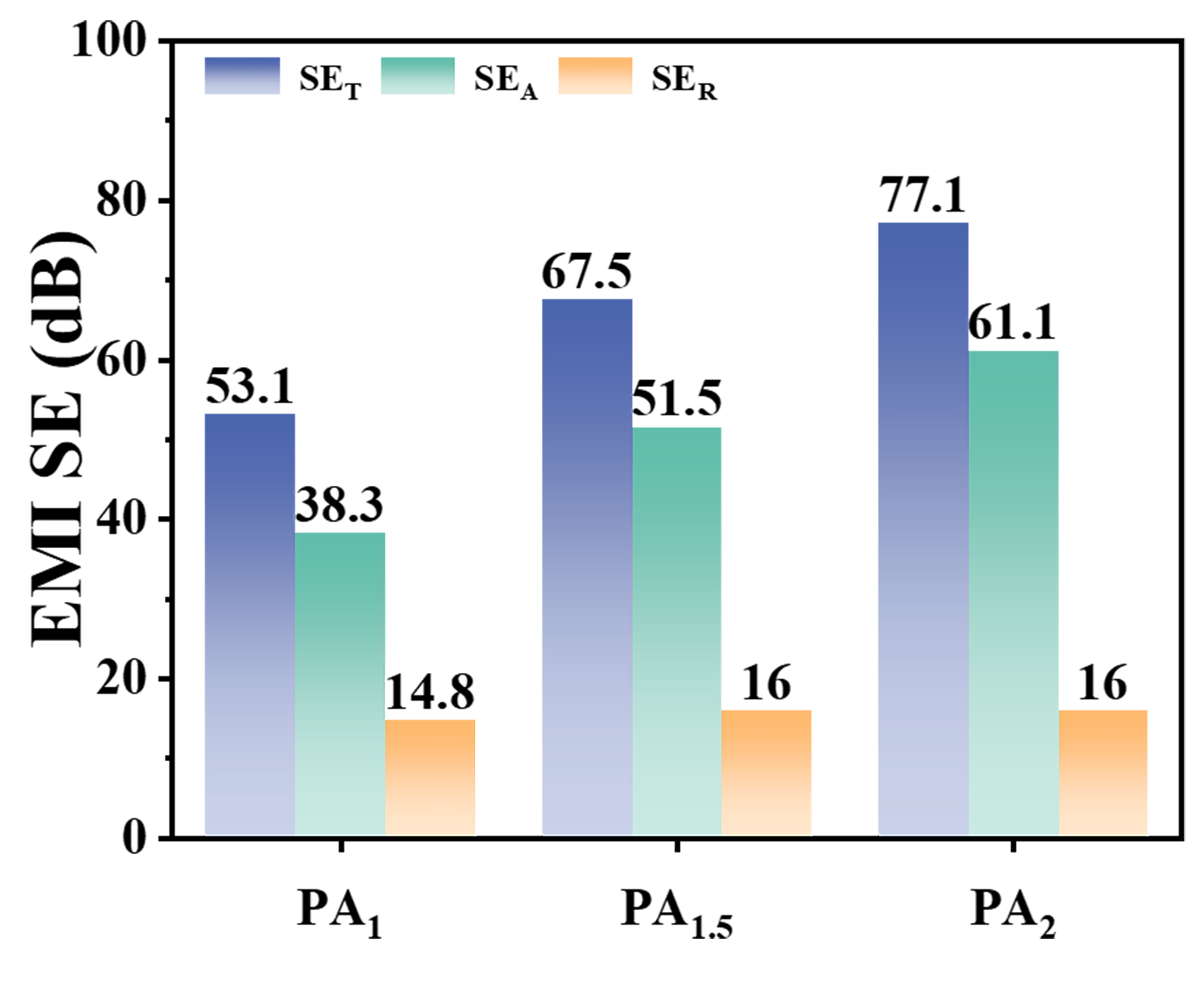
**

**Fig. S12** EMI SE of PA_y_


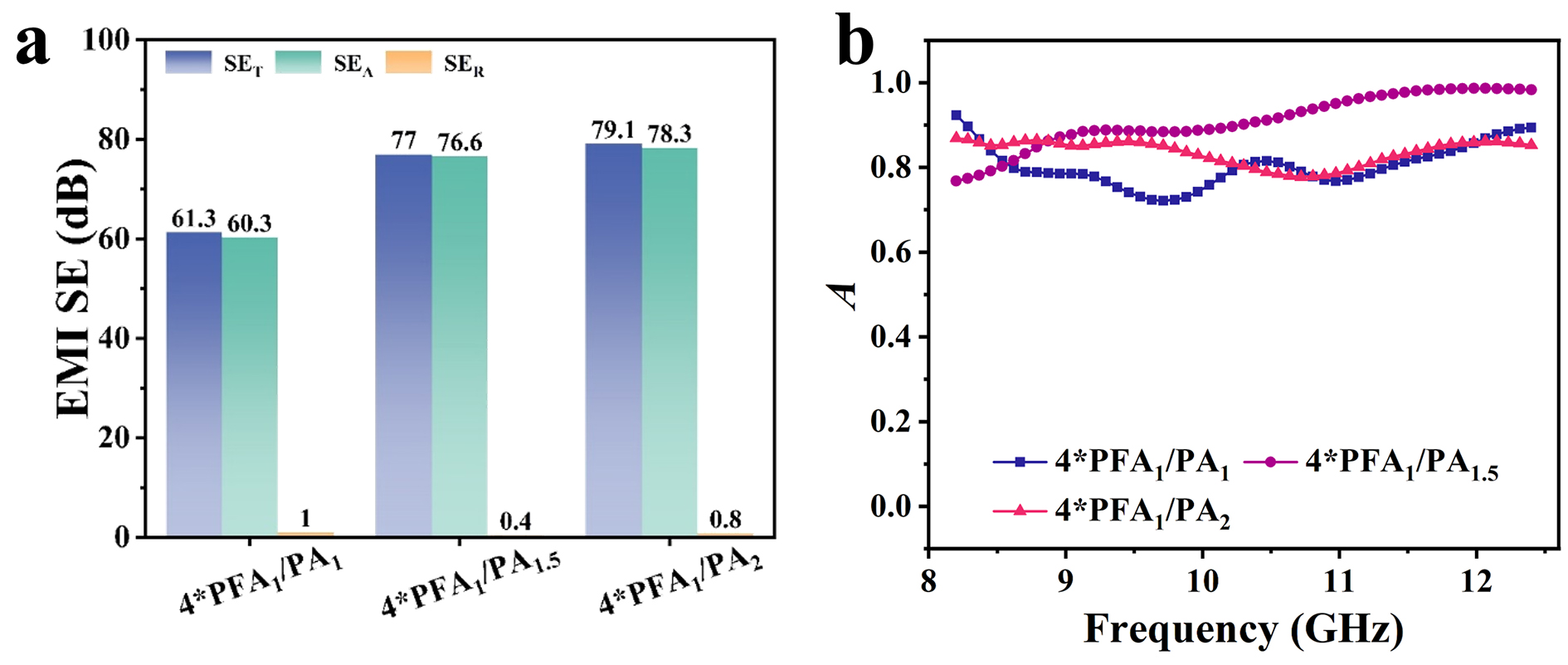


**Fig. S13** (**a**) EMI SE and (**b**) A of 4*PFA_1_/PA_y_


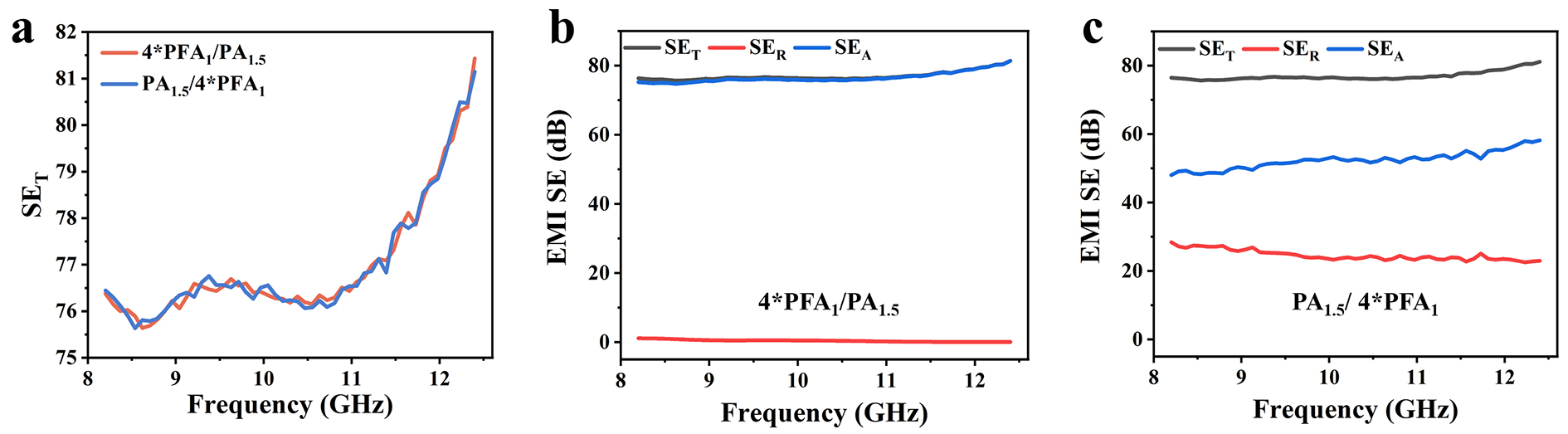


**Fig. S14** (**a**) SE_T_ curves of 4*PFA_1_/PA_1.5_ and PA_1.5_/4*PFA_1_ nonwoven fabrics; EMI SE curves of (**b**) 4*PFA_1_/PA_1.5_ and (**c**) PA_1.5_/4*PFA_1_ nonwoven fabrics


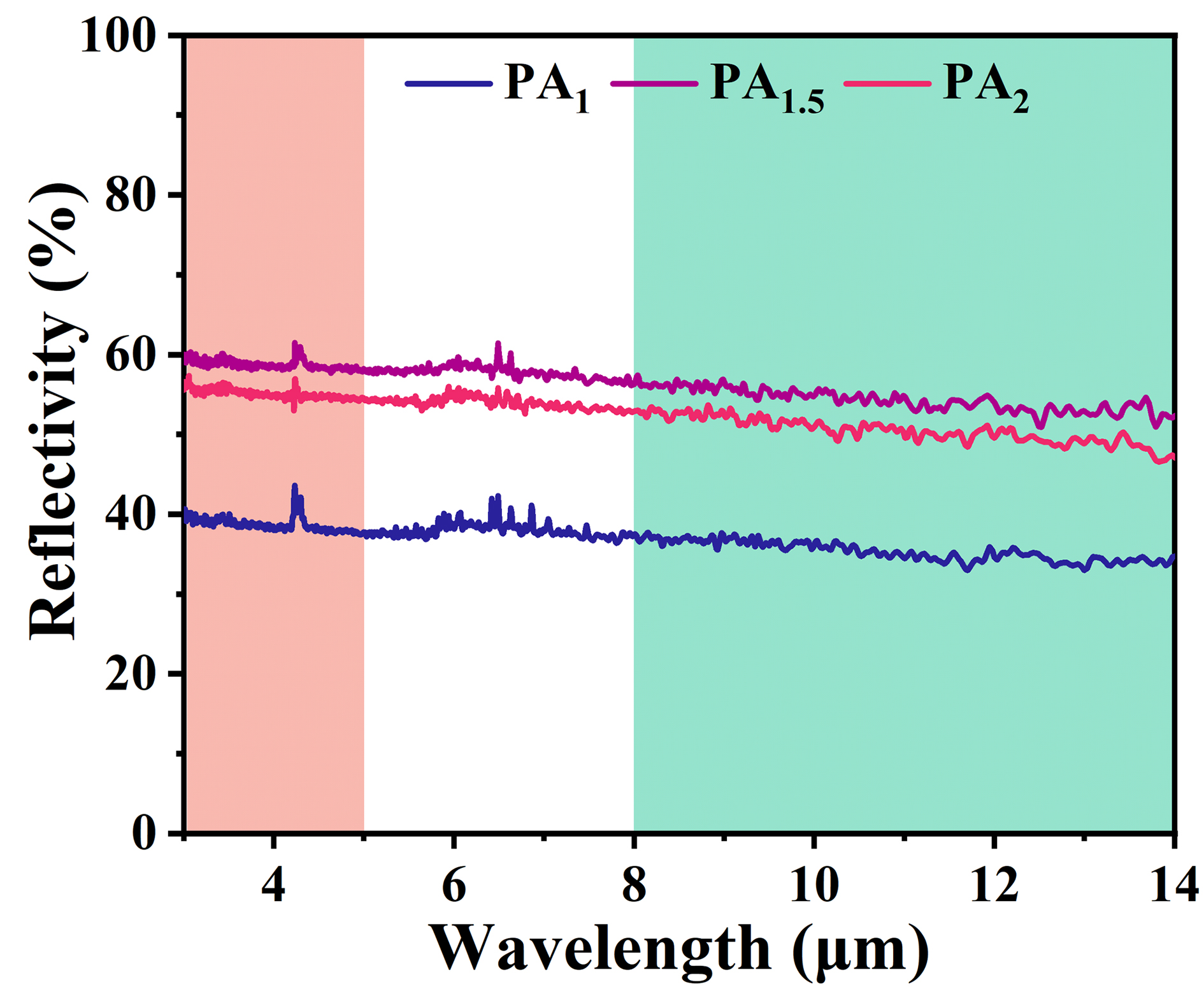


**Fig. S15** IR-reflectivity curves of PA_y_


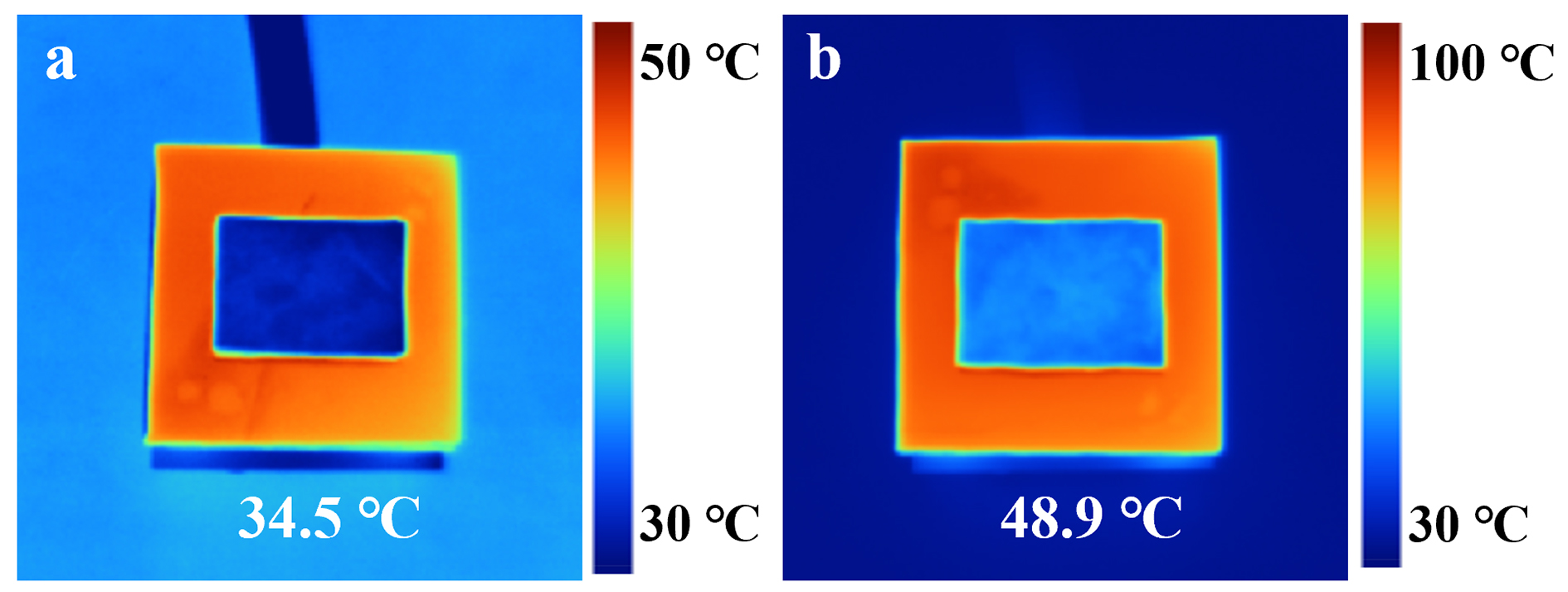


**Fig. S16** IR photograph of 4*PFA_1_/PA_1.5_ at (**a**) 50 °C and (**b**) 100 °C

**Table S1** Comparison for EMI shielding performances of polymer-based materials in X-band

| **Samples** | **Density**  **(g cm^-3^)** | **Thickness (mm)** | **SE_T_**  **(dB)** | **SE_R_**  **(dB)** | **A** | **R** | **Refs.** |
| --- | --- | --- | --- | --- | --- | --- | --- |
| Ni/TPU/CIP | / | 0.5 | 48 | 2.3 | 0.6 | 0.4 | [3] |
| NR/EPMS/CNT | 0.5 | 2 | 44.2 | 2.5 | 0.57 | 0.43 | [51] |
| CS-MWCNTs | 0.01 | 4.9 | 64 | 5.4 | 0.28 | 0.72 | [52] |
| PEBA/CNS | / | 15 | 33 | 0.32 | 0.929 | 0.071 | [53] |
| A4U6C4 | 1.53 | 3.64 | 78.6 | 0.27 | 0.94 | 0.06 | [54] |
| CrO_2_@G-LM | 0.5 | 3.64 | 63.8 | 0.27 | 0.88 | 0.12 | [55] |
| MXene/PEDOT:PSS | 0.01 | 5 | 52 | 3 | 0.65 | 0.35 | [56] |
| SiO_2_/CNTs/PI-AgNWs/CNF | 0.06 | 10 | 110 | 0.023 | 0.995 | 0.005 | [57] |
| CoFe_2_O_4_@MXene/CNTAgNWs/CNF | 1.64 | 0.1 | 87.8 | 5.6 | 0.28 | 0.72 | [58] |
| PCPES/Cu | 0.29 | 0.3 | 59.7 | 9.7 | 0.27 | 0.73 | [30] |
| 4*PFA_1_/PA_1.5_ | 0.17 | 2.46 | 77 | 0.4 | 0.91 | 0.09 | This work |
